# A genome assembly of decaploid *Houttuynia cordata* provides insights into the evolution of *Houttuynia* and the biosynthesis of alkaloids

**DOI:** 10.1093/hr/uhae203

**Published:** 2024-07-30

**Authors:** Peng Huang, Zhu Li, Huan Wang, Jinqiang Huang, Guifeng Tan, Yue Fu, Xiubin Liu, Shang Zheng, Peng Xu, Mengshan Sun, Jianguo Zeng

**Affiliations:** Hunan Key Laboratory of Traditional Chinese Veterinary Medicine, Hunan Agricultural University, Changsha 410128, Hunan, China; College of Veterinary Medicine, Hunan Agricultural University, Changsha 410128, Hunan, China; Traditional Chinese Medicine Breeding Center of Yuelushan Laboratory, Changsha 410128, Hunan, China; College of Animal Science and Technology, Hunan Agricultural University, Changsha 410128, Hunan, China; Wuhan Frasergen Bioinformatics Co., Ltd, Wuhan 430075, Hubei, China; College of Horticulture, Hunan Agricultural University, Changsha 410128, Hunan, China; College of Animal Science and Technology, Hunan Agricultural University, Changsha 410128, Hunan, China; College of Animal Science and Technology, Hunan Agricultural University, Changsha 410128, Hunan, China; Hunan Key Laboratory of Traditional Chinese Veterinary Medicine, Hunan Agricultural University, Changsha 410128, Hunan, China; College of Veterinary Medicine, Hunan Agricultural University, Changsha 410128, Hunan, China; Traditional Chinese Medicine Breeding Center of Yuelushan Laboratory, Changsha 410128, Hunan, China; Wuhan Frasergen Bioinformatics Co., Ltd, Wuhan 430075, Hubei, China; Wuhan Frasergen Bioinformatics Co., Ltd, Wuhan 430075, Hubei, China; Hunan Institute of Agricultural Environment and Ecology, Hunan Academy of Agricultural Sciences, Changsha 410125, Hunan, China; Hunan Key Laboratory of Traditional Chinese Veterinary Medicine, Hunan Agricultural University, Changsha 410128, Hunan, China; College of Veterinary Medicine, Hunan Agricultural University, Changsha 410128, Hunan, China; Traditional Chinese Medicine Breeding Center of Yuelushan Laboratory, Changsha 410128, Hunan, China

## Abstract

***Houttuynia cordata*** Thunb., commonly known as yuxingcao in China, is known for its characteristic fishy smell and is widely recognized as an important herb and vegetable in many parts of Asia. However, the lack of genomic information on ***H. cordata*** limits the understanding of its population structure, genetic diversity, and biosynthesis of medicinal compounds. Here we used single-molecule sequencing, Illumina paired-end sequencing, and chromosome conformation capture technology to construct the first chromosome-scale decaploid ***H. cordata*** reference genome. The genome assembly was 2.63 Gb in size, with 1348 contigs and a contig N50 of 21.94 Mb further clustered and ordered into 88 pseudochromosomes based on Hi-C analysis. The results of genome evolution analysis showed that ***H. cordata *** underwent a whole-genome duplication (WGD) event ~17 million years ago, and an additional WGD event occurred 3.3 million years ago, which may be the main factor leading to the high abundance of multiple copies of orthologous genes. Here, transcriptome sequencing across five different tissues revealed significant expansion and distinct expression patterns of key gene families, such as l-amino acid/l-tryptophan decarboxylase and strictosidine synthase, which are essential for the biosynthesis of isoquinoline and indole alkaloids, along with the identification of genes such as *TTM3*, which is critical for root development. This study constructed the first decaploid medicinal plant genome and revealed the genome evolution and polyploidization events of ***H. cordata***.

## Introduction

*Houttuynia cordata* Thunb., commonly known as yuxingcao in China, is a medicinal, aromatic, perennial plant that has been used for treating various disorders in humans and animals since ancient times and is known as the ‘antibiotic plant’ [1]. The aerial parts of *H. cordata*, including the stems and leaves, are commonly used to treat respiratory diseases such as pneumonia and lung infections. It is often used with other medicines to relieve the symptoms of dysentery, colds, fevers, and mumps. Numerous plant-based components, including a variety of substances such as alkaloids, flavonoids, aristolactams, amides, benzenoids, steroids, 5,4-dioxoaporphines, oxoaporphines, and a range of volatile oils, have been discovered and extracted from *H. cordata*. Among these, alkaloids are the predominant compounds. To date, more than 70 alkaloids, mainly aporphine alkaloids but also aristolochoid alkaloids, amides, pyridines and others, have been isolated from *H. cordata* [2, 3]. Additionally, *H. cordata*, recognized as an edible plant, is a component of various cultural diets, and its young stems, leaves, and underground rhizomes are commonly consumed as vegetables.

**Figure 1:**
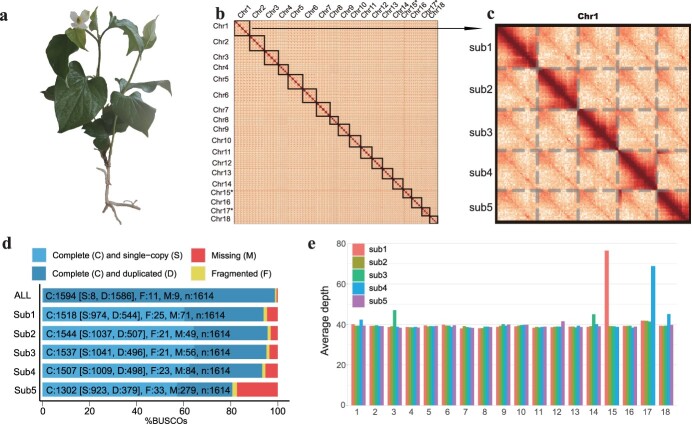
Characterization of the decaploid *H. cordata* genome. **a** Photograph showing the morphology of *H. cordata*. **b** Hi-C interaction heat map for the *H. cordata* genome. **c** Hi-C interaction heat map within chromosome 1. **d** BUSCO assessments in all and sub1–sub5 genomes. **e** HiFi mapping depth of the 88 pseudochromosomes.

*Houttuynia cordata* is the single species of *Houttuynia*, which belongs to the family Saururaceae, the smallest family in the order Piperales within the magnoliid clade [4–7]. The genomes of *Aristolochia fimbriata* (250 Mb, 7 chromosomes), *Aristolochia contorta* (209 Mb, 7 chromosomes), and *Piper nigrum* (769 Mb, 26 chromosomes), all of which belong to the order Piperales, have been published. However, to date no plant genomic data for Saururaceae have been published. The majority of research on this plant has focused primarily on its physiological and biochemical characteristics [8–12], while a limited number of investigations have focused on understanding the genetic variation, population composition, and taxonomic classification of *H. cordata*. Therefore, it is important to perform whole-genome sequencing of *H. cordata* and to analyse the molecular mechanisms underlying its resource diversity at the genomic level.

*Houttuynia cordata* is widely planted in the provinces of Hubei, Hunan, Guizhou, and Sichuan in China. In this study, we collected *H. cordata* plants from Lianghe town in Dangyang (Hubei Province), which is considered the hometown of *H. cordata* in China. Here, we present a high-quality genome of *H. cordata* obtained by integrating Illumina, PacBio and high-throughput chromosome conformation capture (Hi-C) technologies, making the first chromosome-level genome assembly within the Saururaceae family. In addition, we performed transcriptome analysis on five specific parts of *H. cordata* (roots, rhizomes, stems, leaves, and flowers) to specifically investigate the expression levels of genes involved in alkaloid synthesis within the plant. Finally, our analyses comparing genomes and transcriptomes revealed variations in gene expression, evolutionary processes, and gene family sizes associated with alkaloid synthesis. The wealth of new genomic data generated by our study will serve as a vital resource for the genetic improvement of *H. cordata* and for future breeding strategies.

## Results

### Sequencing and assembly

A total of 108.14 Gb (~41× coverage of total genome size) of HiFi reads were obtained with a total of 17.89 kb N50 by utilizing the PacBio Revio platform (Supplementary Data Table S1). To develop a reference genome for *H. cordata* (Fig. 1a), a genome survey was conducted based on *k*-mer frequency before *de novo* genome assembly, which exhibited a peak at a 1:2:3:5 ratio, suggesting a polyploid genome in *H. cordata* (Supplementary Data Fig. S1). The predicted genome size based on *k*-mer statistics was ~2.71 Gb, which was nearly twice the estimated genome size of 1C = 1.57 Gb using flow cytometry analysis (Supplementary Data Fig. S2a), and karyotype analysis revealed 90 chromosomes, indicating a decaploid genome of 2*n* = 10*x* = 90 (Supplementary Data Fig. S2b).

For genome assembly, we employed hifiasm software and obtained a draft genome size of 2.63 Gb, which is consistent with the surveyed genome size. To achieve chromosomal-level assembly, chromosomes of *H. cordata* were crosslinked and sequenced using Hi-C technology (Supplementary Data Table S1). We mapped the contigs to the pseudochromosome level based on the interaction of chromosomes and identified 18 distinct groups (Fig. 1b). Due to the characteristics of polyploidy and high complexity in decaploid species, producing high-quality draft assemblies is usually challenging. Further analysis based on information from the Hi-C heat map and collinearity detected multiple elusive switch errors, significant haplotype collapses, difficult-to-detect inversion errors, and genuine chromosomal exchanges, which were validated or corrected in the *H. cordata* genome assembly (Supplementary Data Figs S3–S6). Finally, each group had five homologous chromosomes (five for 16 haploid assemblies and four for 2 haploids; Fig. 1c), which were named Chr1_sub1, Chr1_sub2, Chr1_sub3, Chr1_sub4, Chr1_sub5 to Chr18_sub1, Chr18_sub2, Chr18_sub3, Chr18_sub4, and Chr18_sub5, for a total of 88 chromosomes, with sizes ranging from 11.34 to 39.59 Mb. The total size was 2.40 Gb, with a contig N50 of 19.83 Mb, representing 91.23% of the genome size (Table 1).

**Table 1:** Assembly statistics of *H. cordata*.

Assembly	*H. cordata*
Estimated size (Gb)	2.71
Assembly length (Gb)	2.63
Number of contigs	1446
Contig N50 (Mb)	19.83
Anchor ratio (%)	91.23%
HiFi read mapping rate (%)	99.77%
HiFi read coverage (%)	99.97%
BUSCO (%)	98.80%
LTR index	13.21
QV	70.50

To assess the quality of the assembled chromosomes, further analysis revealed that 99.77% of the HiFi reads were mapped to our genome assembly (Supplementary Data Table S2). At the whole-genome level, Benchmarking Universal Single-Copy Orthologs (BUSCO) evaluation revealed that 98.80% of the 1614 gene sets were complete BUSCO genes (Fig. 1d, Supplementary Data Table S3), implying that our assembly was complete. Assembly completeness and contiguity were also verified using the long terminal repeat (LTR) assembly index (LAI). The calculated LAI was 13.21, and the genomic QV was 70.50. Finally, these results suggest that the genome assembly of *H. cordata* was very thorough and precise.

The length of our assembled genome is almost twice that predicted by the 1C of the flow cytometry assay and comparable to the size of the full genome survey, indicating that our assembly can be haplotype-resolved. Interestingly, only 88 chromosomes were assembled, which is inconsistent with the karyotyping findings for the 90 chromosomes (Supplementary Data Fig. S2b). We investigated the sequence depth of the assembled genome by mapping HiFi reads to *H. cordata* and found that while most regions were ~40× deep, some regions had a depth of 80×. This result prompted us to speculate that one chromosome in our assembled genome may represent the sequence of two homologous chromosomes in *H. cordata* somatic cells. Specifically, we found that the depth of Chr15_sub1 and Chr17_sub4 was twice that of the other chromosomes (Fig. 1e). The 88 chromosomes we assembled contain sequence information for 90 chromosomes in *H. cordata* somatic cells, as Chr15_sub1 and Chr17_sub4 represent two homologous chromosomes.

### Genome annotation

Annotation of various sequence features of the *H. cordata* genome, including repetitive sequences, protein-coding genes, and non-coding RNA, was performed. Repeat sequence annotation revealed different types of transposable elements, totalling 1.46 Gb (55.52% of the total genome), with LTR elements being the most abundant (Supplementary Data Table S4). Non-coding RNA annotation revealed 504 miRNAs, 4744 tRNAs, 2386 rRNAs, and 893 snRNAs (Supplementary Data Table S5). Finally, 139 087 protein-coding genes were identified using a combination of homology-based annotation, *ab initio* prediction, and transcriptome-based prediction methods, with an average gene length of 5402 bp and an average coding sequence length of 1270 bp, including an average of five exons per gene.

**Figure 2:**
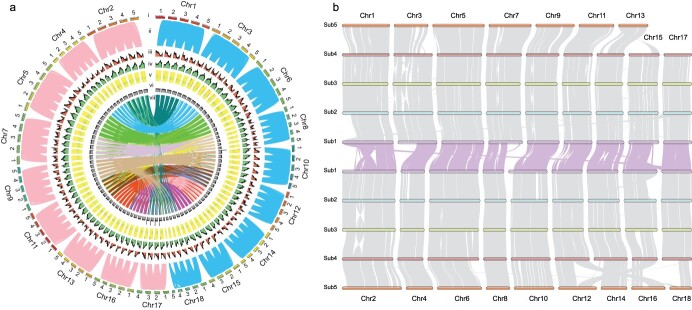
Whole-genome comparisons within *H. cordata*. **a** Circos diagram depicting the relationships of chromosomes between the A and B subgenomes of *H. cordata*. The scale for the chromosomes (outer bars) is in megabases. (i) schematic representation of the 88 chromosome sequences; (ii) synteny gene blocks within subgenomes; (iii) density of protein-coding genes; (iv) repeat coverage; (v) non-coding RNAs; (vi) GC contents; (vii) links inside the circle show intersubgenome syntenic gene pairs. **b** Syntenic gene pair relationships within the *H. cordata* genome.

To assess the accuracy of protein-coding gene annotation, our annotated gene dataset contained 99.4% of the 1614 complete BUSCOs (Supplementary Data Table S3). The assessment of gene density and repeat coverage across the 88 chromosomes of the *H. cordata* genome (Fig. 2a) showed that regions with low gene density typically had high repeat content. A total of 97.78% of the protein-coding genes (136 006 out of 139 087) were homologous to known genes (Supplementary Data Table S6). Moreover, comparative analysis of protein-coding genes from related species (*Macleaya cordata*, *Zingiber officinale*, *P. nigrum*, *A. fimbriata*, and *A. contorta*) was performed. The results indicated that the characteristics of *H. cordata* coding genes, coding sequences, exons, and introns were generally consistent with those of other species (Supplementary Data Fig. S7).

### The assembled *H. cordata* genome can be phased into subgenomes

Our karyotype results are consistent with populations of 2*n* = 10*x* = 90 (Supplementary Data Fig. S2b), indicating that the cardinal number of chromosomes is 9. Further evaluation of the *k*-mer distribution revealed four peaks near 39, 78, 118, and 197, with a ratio of nearly 1:2:3:5, which further supported the finding that *H. cordata* is a decaploid species (Supplementary Data Fig. S1). We also found clear evidence of decaploidy during the Hi-C ordering process, and, as mentioned earlier, the heat map clearly demonstrated the clustering of the pseudochromosomes into 18 homoeologous groups (Fig. 1b), where there was a clear interaction signal between the chromosomes inside the group, which are homologous chromosomes (Fig. 2a, layer i of the circle diagram). Since the chromosome cardinal number is 9 and the Hi-C heat map shows 18 groups, we speculate that there is likely a relationship of homologous chromosomes between each pair of groups, but we found few collinearities at the genome base level (Supplementary Data Fig. S8), which made it impossible to distinguish the correspondence between groups. However, the satisfactory collinearity observed at the gene level further supported the homologous relationship between the nine pairs of groups (Fig. 2a, layer vii of the circle diagram), which was consistent with the karyotyping results for 2*n* = 10*x* = 90 and *x* = 9. That is, the five chromosomes (sub1–sub5) within the 18 groups are homologous chromosomes, and the groups are heterologous chromosome pairs, where Chr1 and Chr2 are heterologous chromosomes, Chr3 and Chr4 are heterologous chromosomes, and Chr17 and Chr18 are paired (Fig. 2b). A total of nine pairs of heterologous chromosomes indicate that nine groups are derived from one parent. The other nine groups were derived from another parent, confirming that the chromosome base number was 9, and the results confirmed that *H. cordata* was a decaploid. An intriguing result from the Smudgeplot analysis suggested that the genome structure of *H. cordata* may be AAAAB (Supplementary Data Fig. S9), i.e. a decaploid genome with both subgenome A and subgenome B. Consequently, we propose that *H. cordata* may be an AAAAABBBBB autoallopolyploid. Since the progenitor genomes of *H. cordata* remain unknown, we reconstructed the phylogenetic relationship between the two homologous chromosomes and two outgroups of *P. nigrum* and *A. fimbriata* to attempt to classify subgenome A and subgenome B by branch length (Supplementary Data Fig. S10). We classified Chr1, Chr3, Chr6, Chr8, Chr10, Chr12, Chr14, Chr15, and Chr18 as subgenome A and Chr2, Chr4, Chr5, Chr7, Chr9, Chr11, Chr13, Chr16, and Chr17 as subgenome B. As a result, the assembled chromosomes were labelled 1A1–1A5 to 9A1–9A5 and 1B1–1B5 to 9B1–9B5. The subA and subB genomes were 1.18 and 1.23 Gb in size, respectively (Supplementary Data Table S7). Consequently, the *H. cordata* A and B subgenomes were assembled.

### Evolutionary scenario of the *H. cordata* genome

**Figure 3:**
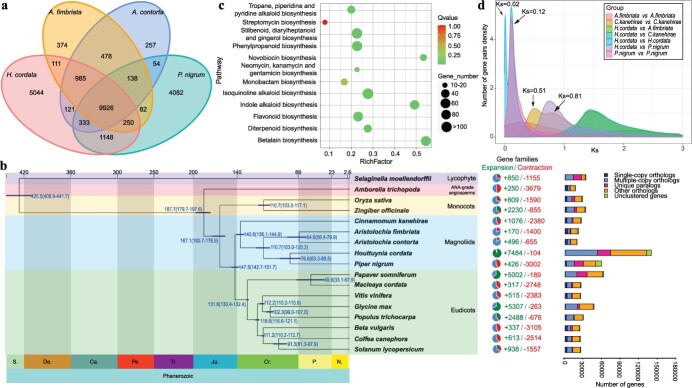
Comparative evolution of *H. cordata* and other species. **a** Venn diagram of numbers of orthologous genes shared among the four Piperaceae species. **b** Phylogenetic relationships and divergence times between *H. cordata* and other species. Divergence times are labelled in blue. The pie charts represent expansions and contractions of gene families. Green, expanded; red, contracted. Clusters of orthologous and paralogous gene families in *H. cordata* and other plant species are shown on the right. **c** Enriched KEGG pathways for expanded genes in *H. cordata*. **d** Distribution of average synonymous substitution values (*K*_s_) between syntenic blocks.

Comparative genomic analyses with 16 other species were performed using *Selaginella moellendorffii* as an outgroup to assess the evolutionary relationships between *H. cordata* and closely related species. When genes were clustered into families based on sequence homology, 9916 families were found to be shared by four species, namely, *H. cordata*, *P. nigrum*, *A. contorta*, and *A. fimbriata*, while 5044 were found to be unique to the *H. cordata* genome (Fig. 3a). These unique gene families are thought to be involved in secondary metabolic synthesis, and these metabolic processes may be related to the abundant active components in *H. cordata.* The phylogenomic analysis placed monocots as a sister clade to the magnoliid + eudicot clade with 100% bootstrap support (Supplementary Data Fig. S11). This result is in agreement with results from a phylotranscriptomic analysis of 92 streptophytes and land plants [13], an angiosperm phylogeny of 26 species [14], a phylogenomic analysis of the stout camphor tree [15], and a phylogenomic analysis of 1000 plant transcriptomes [16]. Of course, the taxonomic status of magnolia plants is controversial and has been discussed in many studies [17, 18]; we will not go into the details here. Furthermore, our results showed that *H. cordata*, *P. nigrum*, *A. contorta*, and *A. fimbriata* formed a clade (Piperaceae) that was expected [5], and *H. cordata* was most closely related to *P. nigrum*, followed by *Aristolochia*. The study also showed that *H. cordata* and *P. nigrum* diverged from their common ancestor ~76.6 Mya (Fig. 3b).

The evolution of gene families (i.e. expansion and/or contraction) is considered to play an essential role in adaptive diversification [19]. We identified 7484 genes that expanded after the divergence of *H. cordata* from *P. nigrum* by analysing gene families with sequence homology. The genes whose expression increased significantly (*Q* value <0.05) were enriched in KEGG terms associated with sesquiterpene synthase activity, sesquiterpene metabolic process, sesquiterpenoid and triterpenoid biosynthesis, phenylpropanoid biosynthesis, tropane, piperidine, and pyridine alkaloid biosynthesis, isoquinoline alkaloid biosynthesis, flavonoid biosynthesis, diterpenoid biosynthesis, and monoterpenoid biosynthesis (Fig. 3c, Supplementary Data Table S8). These results indicated that *H. cordata* was rich in alkaloids, flavonoids, volatile oils, and other chemical components with medicinal value, which was closely related to the enrichment results of the expanded gene families.

### Analysis of whole-genome duplication

To assess the whole-genome duplication (WGD) events in *H. cordata*, we performed a comparative analysis of a range of species, including *Cinnamomum camphora*, a representative species of the Magnoliaceae family, which has undergone two rounds of recent WGD [15]; *A. fimbriata* (which has not undergone WGD events) [20]; and *P. nigrum* (in which a recent WGD event occurred ∼17 Mya) [21]. We first determined the intragenomic gene collinearity, which identified 199 329 gene pairs in 3753 homologous blocks of the *H. cordata* genome. Following the same procedure, we detected 586, 48 and 312 homologous blocks in the *P. nigrum*, *A. fimbriata*, and *C. camphora* genomes, which contained 12 281, 237 and 4397 collinear gene pairs, respectively. Thus, the *H. cordata* genome contained considerably more homologous gene pairs than the genomes of all of the other three species, suggesting the occurrence of one or more additional WGD events (Supplementary Data Table S9).

Specifically, the investigation of collinear orthologues between *H. cordata* and *P. nigrum* allowed us to preliminarily explore the genomic features and gene duplication phenomena in the Piperaceae family. There were 8431 homologous blocks involving 139 643 collinear gene pairs between the *H. cordata* and *P. nigrum* genomes, of which 75 111 (54.00%) *H. cordata* genes have at least one syntenic gene in *P. nigrum*, and conversely, 21 492 (36.56%) *P. nigrum* genes are syntenic with *H. cordata*. Among these, ~20 052 (93.30%) *P. nigrum* genes have more than two syntenic genes in *H. cordata*, which presumably resulted from segmental, tandem, or single-gene duplications that occurred in *H. cordata* after its divergence from *P. nigrum*. By comparing the genomes of *H. cordata* and *P. nigrum*, several chromosome duplication events in *H. cordata* were identified. Most *P. nigrum* blocks correspond to 10 copies in *H. cordata* (Supplementary Data Fig. S12a and b). Additionally, one *H. cordata* block corresponds to multiple *P. nigrum* blocks. For example, the common ancestor’s chromosomes PnChr25 and PnChr13 fused into one chromosome, Chr3A/Chr3B, in *H. cordata*. Similarly, PnChr9 and PnChr1 also fused into one corresponding chromosome, Chr3A/Chr3B. Furthermore, PnChr19 and PnChr20 correspond to *H. cordata*’s Chr3A/Chr3B chromosomes. These fusions likely occurred after the recent WGD in *H. cordata*, resulting in Chr3A/Chr3B aligning with four *P. nigrum* chromosomes (Supplementary Data Fig. S12c).

Our synonymous substitution rate (*K*_s_) distributions show that *C. camphora* has two *K*_s_ peaks at 0.51 and 0.81, respectively; *P. nigrum* has a *K*_s_ peak at 0.12, and *A. fimbriata* has no *K*_s_ peak, consistent with the findings of previous reports [15, 20, 21]. For *H. cordata*, there was a peak at *K*_s_ ~ 0.12, which is consistent with that of *P. nigrum*, indicating that both *H. cordata* and *P. nigrum* experienced a WGD event ~17 Mya. Additionally, a clear peak at a *K*_s_ of 0.02 was observed, implying an additional recent WGD event ~3.3 Mya (Fig. 3d).

We analysed the origins of duplicate genes in *H. cordata*. The results indicate that WGD/segmental duplication is the predominant type of gene duplication (91.95%, 127 891) compared with the other three types: dispersed duplication (3.05%, 4237), tandem duplication (3.03%, 4215), and proximal duplication (1.26%, 1753). Additionally, we extracted the haploid genome of *H. cordata* for similar analysis (18 chromosomes of the sub1 genome) and found that WGD/segmental duplication is also the most prevalent type (79.99%). *Houttuynia cordata* exhibits the highest proportion of WGD/segmental duplication origin compared with the other four investigated taxa (Supplementary Data Fig. S13).

To find out if genes of WGD duplication origins have biological function preference, functional enrichment analysis was carried out. Genes created through WGD/segmental duplication of the *H. cordata* haplotype genome were enriched with KEGG terms like ‘flavonoid biosynthesis’, ‘plant–pathogen interaction’, ‘cellular senescence’, ‘MAPK signalling pathway–plant’, and ‘photosynthesis’ (Supplementary Data Fig. S14).

**Figure 4:**
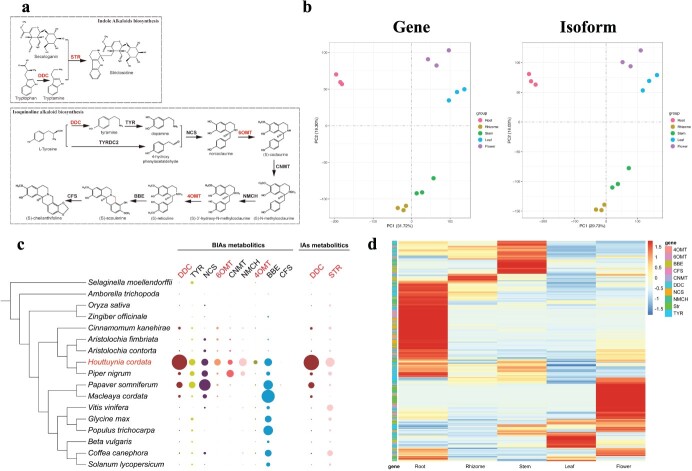
**a** Biosynthetic pathways of IAs and IQAs (significantly enriched gene families are marked in red). **b** PCA plot at the gene and isoform levels in root, rhizomes, stems, leaves, and flowers of *H. cordata*, with different parts being distinguished by different colours: red for roots, brown for rhizomes, green for stems, blue for leaves, and purple for flowers. **c** The left half represents the evolutionary relationship between *H. cordata* and other species with closer relationships in the phylogenetic tree. The right half represents the expression of *4′OMT*, *6OMT*, *BBE*, *CNMT*, *DDC*, *NCS*, *TYR*, *CFS*, and other genes in different species. Circle size indicates the number of gene families, and different genes are represented by different colours. Larger circles represent a higher number of gene families. Gene names marked in red indicate significant expansion of gene families compared with other species. **d** Expression of multiple enzymes catalysing the synthesis of IQAs and IAs in five parts of *H. cordata*.

### Gene family expansion in alkaloid biosynthesis and root development

Alkaloids, a class of nitrogen-containing alkaline organic compounds found in organisms, have a high level of biological activity, making them one of the most important active ingredients in herbal medicines [22–24]. Isoquinoline alkaloids (IQAs) and indole alkaloids (IAs) play exceptional roles in the synthesis of alkaloid compounds, mainly due to their abundant biological activities and wide range of applications. To gain a deeper understanding of the synthesis mechanism of IQAs and IAs in *H. cordata*, we performed transcriptome sequencing analysis of five different tissues (roots, rhizomes, stems, leaves, and flowers) and analysed a number of key gene families involved in the synthesis of IQAs and IAs (Fig. 4a). PCA of the transcriptomic data from different *H. cordata* tissues revealed significant differences among the samples (Fig. 4b). The aromatic l-amino acid/l-tryptophan decarboxylase (*DDC*) gene family, which is crucial for the biosynthesis of IQAs, significantly expanded in *H. cordata* according to comparative genomic results. In the *H. cordata* genome, 102 *DDC* gene homologues were identified, significantly exceeding the 32 and 14 found in *Papaver somniferum* and *Macleaya cordata*, respectively. In addition, the 6-*O*-methyltransferase (*6OMT*) and 4-*O*-methyltransferase (*4OMT*) gene families in *H. cordata* have undergone considerable expansion, with counts of 21 and 34, respectively, far exceeding those in *P. somniferum* and *M. cordata*. *DDC* and strictosidine synthase (*STR*) are crucial for the synthesis of IAs, with the *STR* gene family showing significant expansion (Fig. 4c). In particular, 47 *STR* gene homologues were identified in the genome of *H. cordata*, while 30 and 33 *STR* gene homologues were identified in *Coffea canephora* and *Vitis vinifera*, respectively. Using homologous alignment and Pfam database searches, we meticulously analysed the expression patterns of significantly enriched gene families within the *H. cordata* transcriptome, resulting in the construction of heat maps of transcript expression across different tissues (Fig. 4d, Supplementary Data Figs S15 and S16, Supplementary Data Tables S10 and S11). This exploration of candidate genes provides a fundamental platform for advancing research into the synthesis mechanisms of IQAs in *H. cordata* and identifies potential targets for increasing the levels of IQAs in this species. Additionally, due to the well-developed rhizome and root system of *H. cordata*, we identified nine homologues of triphosphate tunnel metalloenzyme 3 (*TTM3*), a gene closely associated with root structure formation and growth, in its genome. This number significantly exceeds those found in other species, such as *P. nigrum* (four), *M. cordata* (three) and *P. somniferum* (two), as shown in Supplementary Data Fig. S17a. Transcriptome analysis revealed that 60% (6 out of 10) of the *TTM3 *genes were highly expressed in rhizomes, 20% (2 out of 10) in roots, and one each in leaves and flowers, as shown in Supplementary Data Fig. S17b, highlighting the importance of these genes in the development of the root and rhizome systems of *H. cordata*.

## Discussion

*Houttuynia cordata*, an important traditional Chinese medicine, is widely distributed across East Asia. It has attracted increasing attention for its unique flavour and pharmacological value [25]. However, the limited genomic resources available for *H. cordata* have restricted the understanding of its essential agronomic traits. *De novo* assembly of polyploid genomes is challenging due to the presence of multiple subgenomes, high heterozygosity and complicated assembly of repetitive sequences [26, 27]. In this study, we generated a phased decaploid chromosome-level genome of *H. cordata* from Lianghe town, Dangyang. The genome assembly was evaluated using LAI, QV, and BUSCO analyses, representing the first genome of *Houttuynia* within Magnoliidae, Piperales, Saururaceae, and *Houttuynia*. Unfortunately, due to the lack of progenitor genomes, we used phylogenetic trees constructed based on homologous chromosomes to distinguish subgenomes. We consider this result preliminary and believe there is room for improvement, as it is not fully phased. We also attempted various phasing methods, including WGDI [28], SubPhaser [29], centromere evolutionary tree construction typing [30], LTR insertion time difference [20] and so on, but the results did not strongly correlate with the phylogenetic trees, possibly due to the complexity of the decaploid genome. Additionally, for difficult-to-assemble highly repetitive regions, such as telomeres, we used the plant-specific seven-base telomere repeat sequence (3′-TTTAGGG/5′-CCCTAAA) as a query and found telomeres identified at both ends of 57 chromosomes (20 of which are gap-free T2T pseudomolecules), one end identified in 26 chromosomes, with a total of 140 telomeres identified, indicating that 36 telomeres are missing. This presents a challenge for assembly but is an ideal state for a decaploid. Future efforts are needed to generate more data for full phasing and highly repetitive regions.

Comparative genomic analysis revealed that *H. cordata* experienced two additional WGD events after its divergence from other species (17 and 3.3 Mya), indicating that WGD and polyploidization play major roles in plants adapting to stressful conditions [31, 32] and that these processes additionally promote functional advancement through gene duplication, chromosome rearrangement, and genomic repatterning events [33]. Therefore, the identified WGD events in *H. cordata* may be closely associated with species evolution, diversification, and wide adaptation.

STR and DDC play critical roles in the biosynthesis of IQAs and IAs, two important classes of plant secondary metabolites with significant pharmacological properties [34, 35]. STR is integral to the synthesis of IQAs, catalysing the key step leading to the formation of strictosidine, a common precursor for a wide range of IQAs known for cancer treatment [36, 37]. On the other hand, *DDC* is essential for initiating the biosynthetic pathway of IAs by decarboxylating tryptophan to produce tryptamine, which is then involved in the formation of various IAs, compounds known for their anticancer, antimicrobial and neuroprotective effects [38]. Together, these enzymes facilitate the production of a wide range of bioactive compounds, highlighting their importance in plant biochemistry and their potential for developing novel therapeutic agents from IQAs and IAs. *6OMT* and* 4OMT* are critical enzymes in the biosynthetic pathways of alkaloids, particularly in the production of IQAs. These methyltransferases catalyse the transfer of methyl groups from *S*-adenosylmethionine (SAM) to specific substrates, facilitating the synthesis of various bioactive alkaloids [39]. In particular, *6OMT* is responsible for methylating the sixth carbon position, whereas *4OMT* targets the fourth carbon position on alkaloid molecules, a process that is essential for the formation of their final structures and directly influences their chemical properties, biological activities, and pharmacological potential [40]. The involvement of these enzymes in the methylation steps is crucial for the synthesis of important alkaloids such as scoulerine, berberine, and aconitine, which influence the plant's chemical defence mechanisms and provide medicinal value. Therefore, *6OMT* and *4OMT* play a central role in plant metabolic networks, bridging primary and specialized metabolism and significantly influencing plant adaptability and survival strategies, highlighting their importance in the field of plant secondary metabolism and pharmacognosy research.

In addition, our results showed that the biosynthesis of alkaloids in *H. cordata* occurs mainly in the root tissues. The localization of alkaloids primarily in the roots of *H. cordata* emphasizes their pivotal role in defending against soil-borne pathogens and herbivores. Such a sophisticated mechanism ensures the efficient synthesis and accumulation of bioactive compounds precisely where they are most needed to deter underground pests and diseases, thereby enhancing the plant's survival and adaptability in its natural habitat [41, 42]. Understanding the gene expression patterns within root tissues provides profound insights into the metabolic pathways that facilitate the synthesis of alkaloids, thereby providing valuable information for enhancing the pharmacological potential of *H. cordata* through targeted genetic and metabolic engineering strategies. Considering the diverse biological activities of IQAs and IAs, including their anti-inflammatory, antimicrobial, and anticancer properties, the expansion of this gene family in *H. cordata* may reflect an evolutionary adaptation aimed at enhancing the synthesis of IQAs and IAs to combat pests and diseases in its environment, thereby facilitating better adaptation to complex habitats. The expansion of gene families associated with the biosynthesis of alkaloids, including IQAs and IAs, in *H. cordata* not only offers critical insights for a more profound exploration of the synthesis mechanisms of IQAs and IAs within this plant but also presents potential targets for genetic engineering approaches aimed at enhancing the concentrations of these bioactive compounds. Such augmentation could significantly bolster the development of novel pharmaceuticals and improve plant defence mechanisms.

Previous research has shown that the *TTM3* gene, which is predominantly expressed in the root meristematic zone of *Arabidopsis thaliana*, plays a crucial role in root development, with gene knockout experiments resulting in developmental delays in mutants, highlighting its indispensable role in the formation and growth of root structures [43]. In the tetraploid *Rheum officinale*, a medicinal plant, the *TTM3* gene is thought to be closely linked to the regulation of root development and has undergone significant expansion within its genome [44]. Here, the expansion of the *TTM3* gene family in *H. cordata* may enhance the plant's ability to adapt to its environment, for example by improving the efficiency of nutrient uptake from the soil or increasing resistance to abiotic stresses such as drought and salinity. Consequently, this extension may directly influence the morphogenesis and functionality of *H. cordata* roots, facilitating more effective support of plant growth and reproduction by optimizing root health and development.

There are considerable differences in chromosome number and polyploidy levels among *H. cordata* populations in different regions (2*n* = 24–128), which may be one of the factors affecting yield [7, 45]. This is probably due to the prevalence of cytomixis and the occurrence of meiotic abnormalities during microsporogenesis [13]. This study confirmed that the *H. cordata* samples from Dangyang are decaploid, and these plants exhibit superior growth performance, yield, and nutritional value compared with diploids [46]. Therefore, the chromosomal ploidy of the plants is closely related to the high yield and quality of *H. cordata* in our study. The study of chromosomal variation not only helps elucidate the evolutionary trajectory of plants, acting as a driver of plant evolution, genetic diversity and the formation of new species, but also plays a crucial role in plant genetic improvement and the development of new cultivars [47]. Prior to this study, no genome sequences of decaploid medicinal plants had been reported, with only decaploid pitcher plant genomes documented [48]. The sequencing of the genome will deepen our understanding of the unique alkaloid biosynthesis and chromosome ploidy of the decaploid *H. cordata*.

## Materials and methods

### Samples and DNA sequencing

Samples of *H. cordata* were gathered from its cultivation site in Lianghe town, Dangyang, Hubei Province, located at 30°39′36″N, 111°56′19″E. Immediately post-harvest, these specimens were rinsed with distilled water to strip away soil and any external impurities, ensuring the integrity of the plant material for subsequent analysis. To avoid water-related damage or the compromise of genetic integrity, we carefully removed surplus moisture from each sample. The samples were then rapidly frozen in liquid nitrogen, halting metabolic processes and safeguarding the RNA and DNA. For genomic DNA extraction from the leaves, a customized CTAB method [49] was employed, followed by RNase A treatment to eliminate RNA impurities. We assessed the DNA’s quality and volume using a NanoDrop 2000 spectrophotometer and agarose gel electrophoresis, ensuring high fidelity for further analysis.

We prepared the SMRTbell library with the SMRTbell Express Template Prep Kit 2.0, starting with genomic DNA (~15 μg) that was fragmented to ~15 kb using Covaris g-TUBE. Following DNA repair and end preparation, an A-overhang was added, and the SMRTbell adapter was attached at 20°C for 15 h. We then purified the library with 1X AMPure PB magnetic beads. Fragment size distribution was checked with Femto Pulse, and large fragments (>15 kb) were selected using a BluePippin system (Sage Science). We evaluated the prepared library’s quality and volume using Femto Pulse and Qubit fluorometer, respectively. Sequencing was performed on the PacBio Revio system for 24 h at Frasergen Bioinformatics in Wuhan, capturing a full day’s data per SMRT cell.

### Transcriptome library construction and sequencing

We extracted total RNA with TRIzol reagent and ensured its purity and structural integrity using a NanoDrop 2000 spectrophotometer and a Bioanalyzer 2100 system. Agarose gel electrophoresis at 1.5% was employed to check for RNA impurities. mRNA was then isolated using poly-T oligo-attached magnetic beads, and sequencing libraries were created from this mRNA with the VAHTS Universal V6 RNA-seq Library Kit for MGI, incorporating unique indexing for each sample. We quantified and measured the libraries using a Qubit 3.0 Fluorometer and the Bioanalyzer 2100. Finally, sequencing was conducted on the MGI-SEQ 2000 platform at Frasergen Bioinformatics in Wuhan, China, following standard protocols.

### Genome feature estimation

We used Jellyfish (v2.3.05) [50] to count the *k*-mers (*K* = 17) and GCE software (v1.0.2) [51] to analyse these *k*-mers in our sequencing data to infer genome size and heterozygosity. In addition, to determine the genome size of *H. cordata*, *Zea mays* was used as a baseline by flow cytometry [52]. For flow cytometry analysis, nuclei extracted from both species were stained with DAPI. We determined *H. cordata*’s genome size by comparing the 2C peak DNA content ratios to that of *Z. mays*, which has a documented genome size of 2.5 Gb.

### Karyotype analysis

After 0.002 M 8-hydroxyquinoline (8-HQ) treatment for 1 day, we observed mitosis and then fixed the sample in Carnoy’s solution (absolute ethanol:glacial acetic acid; 3:1, v/v) for 30 min before storing it at −20°C. The samples were then broken down with a 2% cellulase and 20% pectinase solution at 37°C for 40 min. Crushing the samples in 45% acetic acid between a slide and cover slip, we quickly plunged them into liquid nitrogen to detach the coverslip. We stained the samples with DAPI (2 μg/ml) in a 50% glycerol solution, selecting the best ones for further analysis. The samples were refixed in Carnoy’s solution at room temperature for 30 min and preserved in absolute ethanol for 2 h. After air-drying the slides for 3 days, each sample received a 10-μl CMA (0.1 mg/mL) stain for an hour and a 10-μl DAPI (1 μl/ml) stain for 30 min. The final step involved mounting the slides with a glycerine and McIlvain buffer mixture at pH 7.0 (1:1, v/v), then storing them in the dark for 3 days [53].

For each slide, images of at least 10 cells were captured with an Axio Cam MRC5 digital camera, utilizing AxioVision 4.8 software from Carl Zeiss Microscopy, Jena, Germany. We compiled the final images using Photoshop CS3 Extended 10.0 by Adobe Systems, San Jose, USA. Chromosome measurements and analysis were conducted using Image Tool 3.0 software, with the morphology identified through the centromere index method outlined by Guerra [54]. Categorization of heterochromatin banding patterns was based on the approach established by Cornélio *et al*. [55].

### Genome assembly with HiFi reads

Utilizing OneSMRT cells on the PacBio Revio platform, we produced 108.14 Gb of HiFi reads, achieving over 99% accuracy and covering the genome 41-fold (Supplementary Data Table S1). These reads were instrumental in assembling the *H. cordata* genome. We employed hifiasm (v0.16.1) [56] for the initial assembly, using its standard settings. For converting sequence graphs from GFA to FASTA format, we utilized gfatools, available at https://github.com/lh3/gfatools.

### Chromosome assignment using Hi-C technology

We employed Trimmomatic (v0.40) [57] to refine high-quality paired-end reads by eliminating low-quality bases and adapter sequences. The processed reads were then mapped to contigs using Juicer [58] (v3, available at https://github.com/aidenlab/juicer) to assess contact frequency. For correcting misjoins, we used 3ddna (v180922) [59] through two iterative rounds (−r2) with the standard settings. We then used oriented scaffolds to construct interaction matrices in Juicer, which were further examined and manually adjusted using Juicebox assembly tools (v1.11.08) [58].

To address the difficulties of assembling polyploid genomes, we have also adopted some strategies to reduce chimeric assembly and enhance the overall quality of the assembly. We first extracted the longest chromosome from each group, totalling 18 chromosomes, and conducted pairwise comparisons using Minimap2 (v2.24, parameter -x asm5) [60]. Subsequently, we used dotPlotly (https://github.com/tpoorten/dotPlotly) to visualize the synteny, preliminarily identifying the homology relationships among the 18 groups at the genome level. After gene annotation (see the detailed description below), we employed JCVI software [github.com/tanghaibao/jcvi/wiki/MCscan-(Python-version)] to determine the homology relationships among the 18 groups at the gene level, confirming that *H. cordata* is a decaploid with an AAAAABBBBB configuration. Additionally, we utilized the collinearity between homologous chromosomes and Hi-C interaction information through Juicebox to integrate the assessment of chromosome assembly quality and integrity (Supplementary Data Figs S3–S6).

We used the branch length of the phylogenetic tree based on the orthologous genes to partition subgenomes. The *P. nigrum* and *A. fimbriata* genomes were used to identify synteny blocks and orthologous gene pairs for each chromosome. Orthologous gene pairs were subjected to multiple sequence alignment and concatenated within each chromosome. Phylogenetic trees were then built based on the concatenated sequences.

### Assessment of assembly quality

We utilized the Minimap2 algorithm (v2.24) [60] to gauge both coverage and completeness of the genome assembly by mapping HiFi reads with the ‘-ax map-hifi’ setting. To further assess the assembly's completeness, we applied BUSCO (v3.0.2) [61] using the embryophyta_odb10 database. Recognizing that repetitive sequences often pose assembly challenges, we calculated the LAI [62] for identified LTR elements within the assembly. Additionally, we evaluated the genome's *k*-mer-based quality (*k* = 19 bp) employing the Merqury pipeline (v1.3) [63] and HiFi reads.

### Repeat annotation

We conducted searches for repetitive sequences, including tandem repeats and transposable elements (TEs), using a two-pronged approach. Initially, Tandem Repeats Finder (TRF, v4.09.1) [64] was applied to mark tandem repeats with specific parameters (2, 7, 7, 80, 10, 50, 2000). Subsequently, TEs were pinpointed through both *de novo* and homology-based methods at the DNA and protein levels. DNA-wise, LTR_FINDER (v1.0.7) [65] pinpointed LTR retrotransposons (LTR-RTs), while RepeatModeler (v2.0.1) [66] built a *de novo* library encapsulating consensus repeats with classification. RepeatMasker (v4.1.2) [67] was then used to align these findings against both a known Repbase TE library [68, 69] and the *de novo* library. Protein-wise, searches within the TE protein database were executed using RepeatProteinMask from the RepeatMasker suite, leveraging the WU-BLASTX engine for comprehensive TE identification.

### Gene annotation

To predict the coding gene structures, we utilized a combination of homologous, *ab initio*, and transcriptome-assisted annotation approaches. For homology-based annotation, we applied tblastn (v2.11.0+) [70] to compare our reference genome with those of related species, including *P. nigrum*, *A. fimbriata*, *A. contorta*, *M. cordata*, and *Z. officinale*. The aligned sequences and proteins were then refined and aligned accurately using Exonerate (v2.4.0) [71]. Augustus (v3.4.0) [72–74] and GlimmerHMM (v3.0.4) [75] facilitated *de novo* annotation. RNA-seq data were analysed using both *de novo* and genome-guided transcriptome assemblies, with HISAT2 (v.2.2.1) [76] for alignment and StringTie (v.2.1.7) [77] for transcript assembly. Additionally, Trinity (v2.8.5) [78] enabled the *de novo* assembly of the transcriptome. We compiled a comprehensive transcriptome database from all RNA-seq and Iso-seq transcripts via the PASA pipeline (v2.4.1) [79]. Integration of the gene predictions into a consolidated gene set was achieved with Maker (v3.01.03) [80]. The PASA pipeline (v2.4.1) [79] was then employed to refine the Maker consensus, adding UTR annotations and models for alternative splicing.

### Functional annotations

Gene functions were deduced by aligning sequences with top matches from databases such as NCBI Nonredundant (NR), Kyoto Encyclopedia of Genes and Genomes (KEGG) [81], Gene Ontology (GO) [82], TrEMBL [83], and Swiss-Prot [83], using Diamond BLASTP (v2.0.7) [84] with an E-value limit of 1E−5. We annotated protein domains using InterProScan (v5.50–84.0) [85], utilizing the comprehensive InterPro [86] database for detailed insights.

### Annotation of non-coding RNA genes

We deployed tRNAscan-SE (v2.0.9) [87] to detect tRNA genes, serving as crucial adapters that connect mRNA’s 3-letter genetic code to the amino acid’s 20-letter sequence in protein synthesis. RNAmmer (v1.2) [88] was tasked with uncovering rRNA sequences. snoRNAs, which are pivotal in guiding RNA modifications, particularly in ribosomal RNAs, tRNAs, and small nuclear RNAs, were examined. Additionally, miRNAs and snRNAs were pinpointed using Infernal (v1.1.2) [89] software, searching the Rfam (v14.6) database [90] under standard settings.

### Gene family and phylogenetic analysis

OrthoFinder2 (v2.5.4) [91] was employed to define protein-coding gene families by clustering proteins across 16 diverse species such as *S. moellendorffii*, *Amborella trichopoda, Oryza sativa*, *Z. officinale*, *P. somniferum*, *Populus trichocarpa*, *Coffea canephora*, *V. vinifera*, *Solanum lycopersicum*, *Glycine max*, *Beta vulgaris*, *M. cordata*, *Cinnamomum kanehirae*, *A. fimbriata*, *A. contorta*, and *P. nigrum*, utilizing ‘-M msa -S diamond’ for analysis. Sequences from *A. contorta* and *C. kanehirae* were sourced from https://genomevolution.org, *P. nigrum* from http://cotton.hzau.edu.cn, and the remainder from NCBI.

Phylogenetic analysis was conducted on 114 low-copy orthologous genes, with selection criteria including being single- or double-copy in select species and strictly single-copy in others. MUSCLE (v3.8.31) [92] aligned the protein sequences, and these alignments guided the concatenation of coding sequences. RAxML (v8.2.12) [93] constructed the phylogenetic tree using the maximum likelihood method, with divergence times refined using TimeTree [94] and further calibrated with R8S (v1.81) [95]. Divergence times were then estimated using MCMCTree from PAML (v4.10.7) [96].

### Gene family expansion and contraction analysis

Utilizing the gene families and a phylogenetic tree with estimated divergence times, CAFÉ [97] analysed gene family dynamics through a random birth and death model. We calculated conditional *P* values for each family, considering those with *P* values under 0.05 as having significant gene gain or loss acceleration. These gene families, either expanded or contracted in *H. cordata*, underwent GO and KEGG enrichment analyses. This process employed a hypergeometric test, with significance determined by a false discovery rate (FDR)-adjusted *P* value (*Q* value) of <0.05.

### Genome synteny and whole-genome duplication

We transformed GFF files of *A. contorta*, *C. kanehirae, P. nigrum*, and *H. cordata* into BED format, leveraging MCscan [available at github.com/tanghaibao/jcvi/wiki/MCscan-(Python-version)] to detect syntenic blocks among these genomes with ‘python3 -m jcvi.compara.catalog ortholog’. Synteny analysis was conducted using ‘python -m jcvi.compara.synteny’, and visualizations of pairwise synteny for the *H. cordata* genome were created using ‘python -m jcvi.graphics karyotype’. For WGD analysis, paralogous and orthologous gene pairs from these syntenic blocks were utilized to compute *K*_s_ values using PAML (v4.10.0) [98] yn00 NG model. Divergence times were derived using the formula *K*_s_/2*r*, where *r* represents the mutation rate (3.02E−9), based on estimates from Cui *et al*. [99]. The distribution of *K*_s_ values facilitated the assessment of potential WGD events within each genome.

The genes of *H. cordata* were classified as singletons, dispersed duplicates, proximal duplicates, tandem duplicates, and segmental/WGD duplicates using the duplicate_gene_classifier module in MCScanX [100] by parsing the all-versus-all BLASTP results.

### Differential gene expression analysis

SOAPnuke [101] (V2.1.0) was employed for raw sequencing data cleanup, with HISAT2 [102] (V2.1.0) mapping the filtered reads to the reference genome. RSEM [103] software quantified the read count for each transcript per sample, facilitating FPKM [104] (fragments per kilobase per million bases) analysis. DESeq2 [105] (V1.22.2) conducted the differential expression analysis, adopting an FDR of <0.05 and a log_2_ fold change threshold of >1 or <−1 for significance screening.

## Supplementary Material

Web_Material_uhae203

## Data Availability

The raw sequence data (PacBio and Hi-C) and whole genome assembly reported in this study have been deposited in the Genome Sequence Archive [106] and Genome Warehouse [107], respectively, at the National Genomics Data Center [108] (https://ngdc.cncb.ac.cn) under BioProject PRJCA024754.
