# Supplementary material for: A genome assembly of decaploid Houttuynia cordata provides insights into the evolution of Houttuynia and the biosynthesis of alkaloids
Source: Hortic Res. 2024 Jul 30;11(9):uhae203. doi: 10.1093/hr/uhae203 (PMC11415239; doi:10.1093/hr/uhae203)
Supplement: Web_Material_uhae203 [file web_material_uhae203.zip › Supplemenatary Figures 1-17.docx]

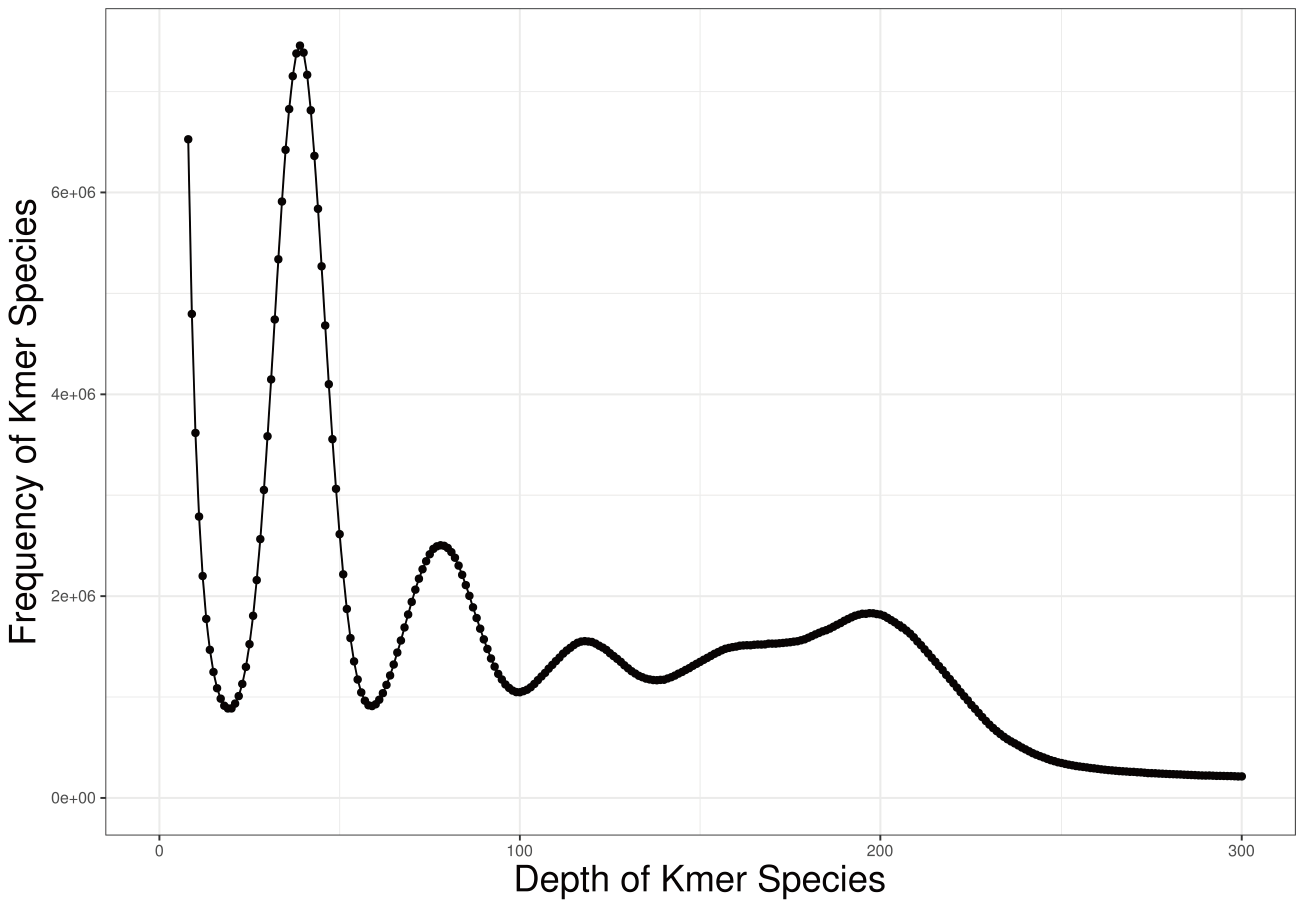


**Figure S1**. Evaluation of *H. cordata* genome size by *k*-mer analysis (k=17).


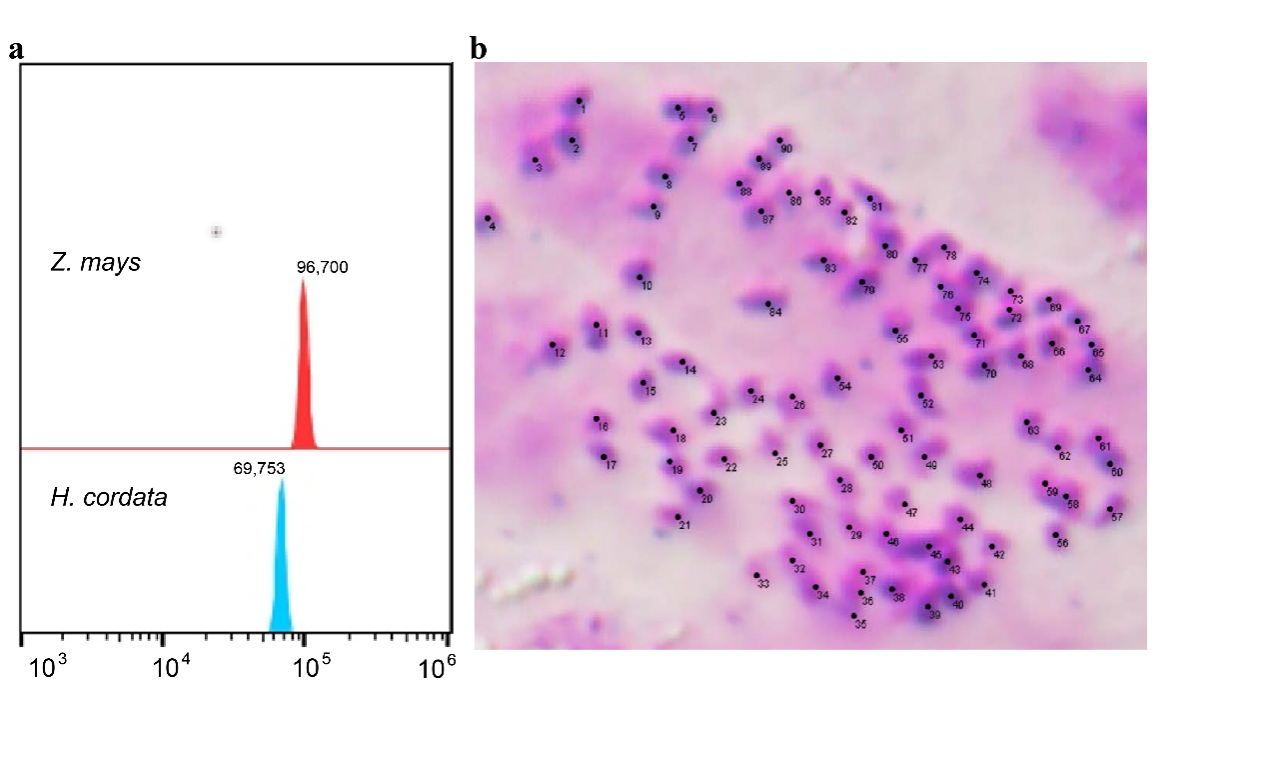


**Figure S2**. (a) Flow cytometry analysis of the genome size of *H. cordata* with Z. mays as internal reference. (b) Chromosome counting for *H. cordata* root tip cell.


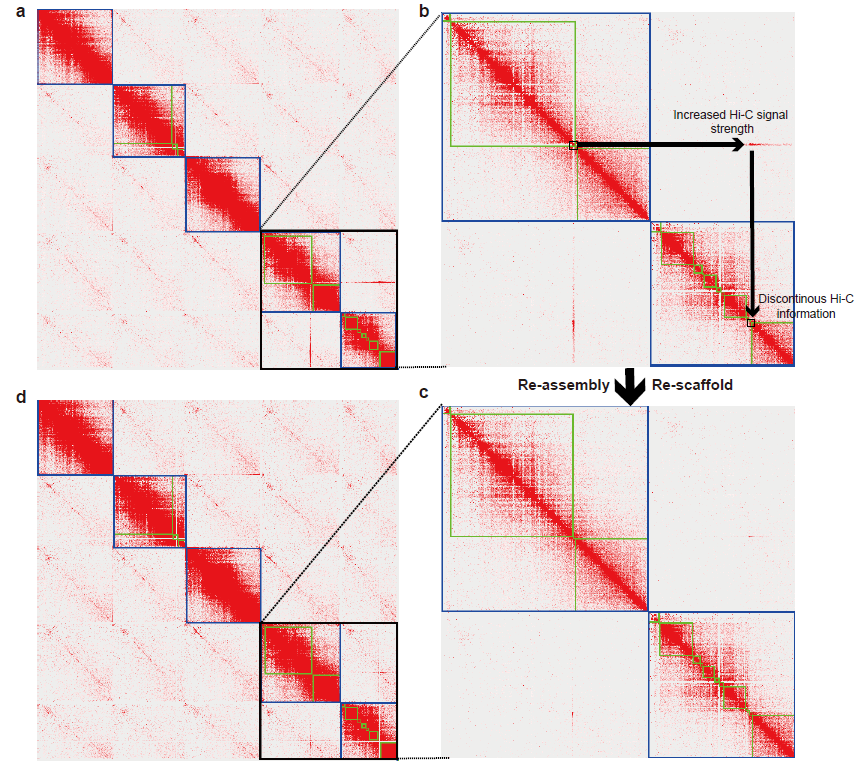


**Figure S3**. The working flow of switch errors detection and reassembly between homologous chromosomes.


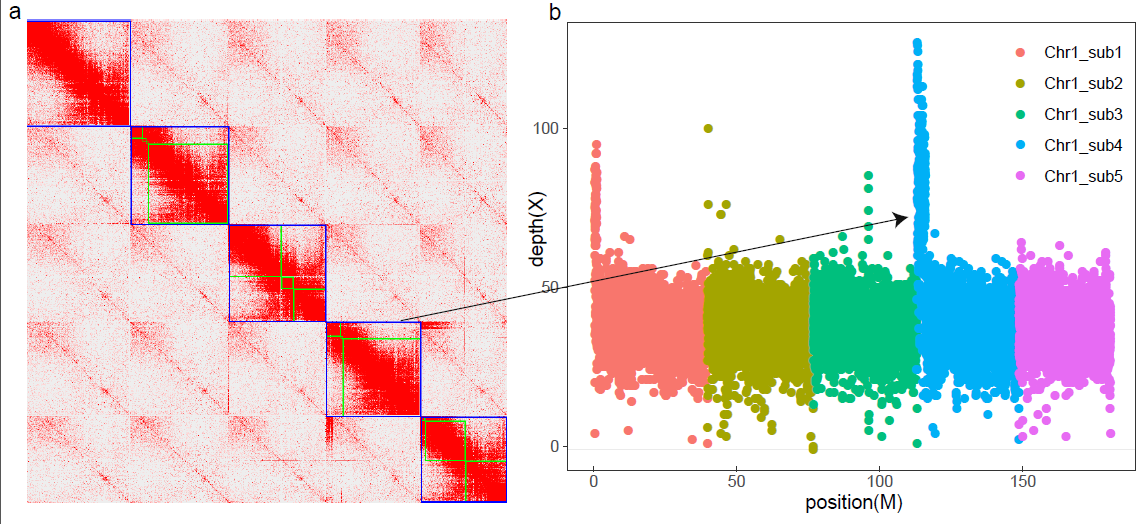


**Figure S4**. Local case for the depth elevation interval in *H.cordata* assemblies: (a) Hi-C interaction in homozygous collapse intervals of Chr1, which dispersed among homologous chromosomes within subgenomes and (b) the corresponding short read mapping depth.


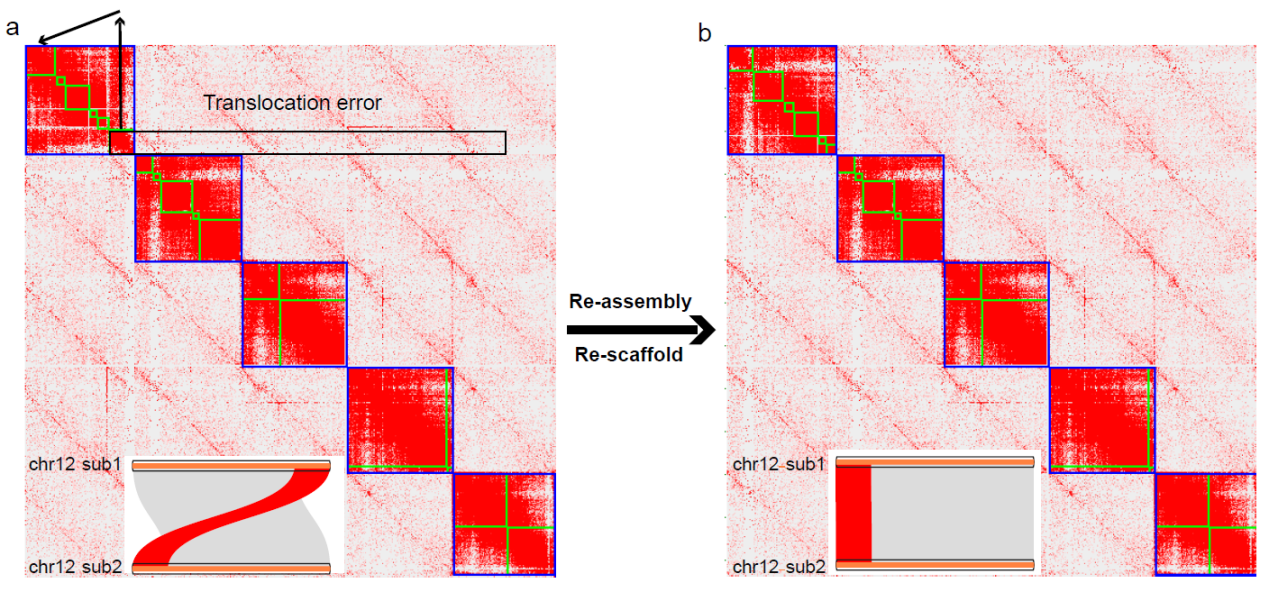


**Figure S5**. Example of Hi-C assembly adjustment based on collinearity of homologous chromosomes: (a) Assembly errors could not be detected based solely on the Hi-C heatmap, but clear inversions were observed in sub1 and sub2 of Chr12, suggesting a possible assembly error; (b) After adjusting the Hi-C heatmap, the result appeared more reasonable, and the sequence inversions were no longer present.


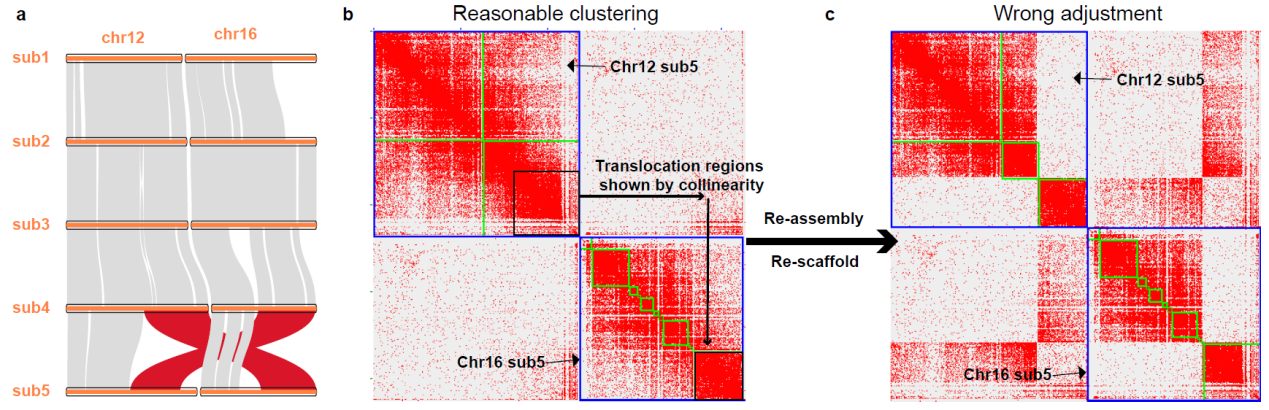


**Figure S6**. Example of Hi-C assembly that does not require adjustment based on collinearity of homologous chromosomes: (a) Collinearity indicates a chromosomal translocation between Chr12 sub5 and Chr16 sub5; (b) The Hi-C heatmap confirms the existence of this homologous translocation; (c) The Hi-C heatmap demonstrates that the original assembly is correct and reasonable, and does not support adjusting Chr12 sub5 and Chr16 sub5 based on the collinearity results.


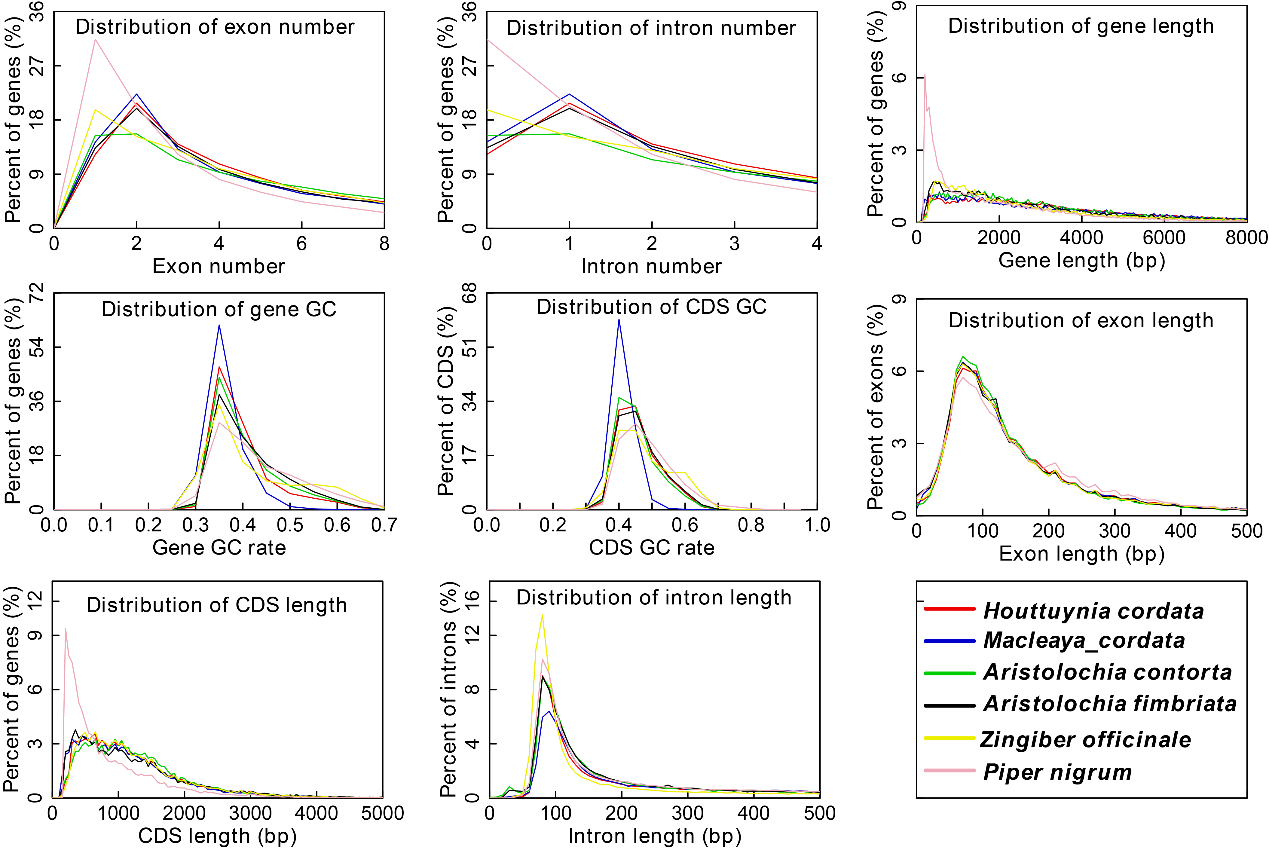


**Figure S7**. Distribution of exon number, intron number, gene GC, coding sequence (CDS) GC, gene length, CDS length, exon length, and intron length in the *H. cordata* genome as compared to *M.cordata*, *A. contorta*, *A. fimbriata*, *Z. officinale*, and *P. nigrum*.


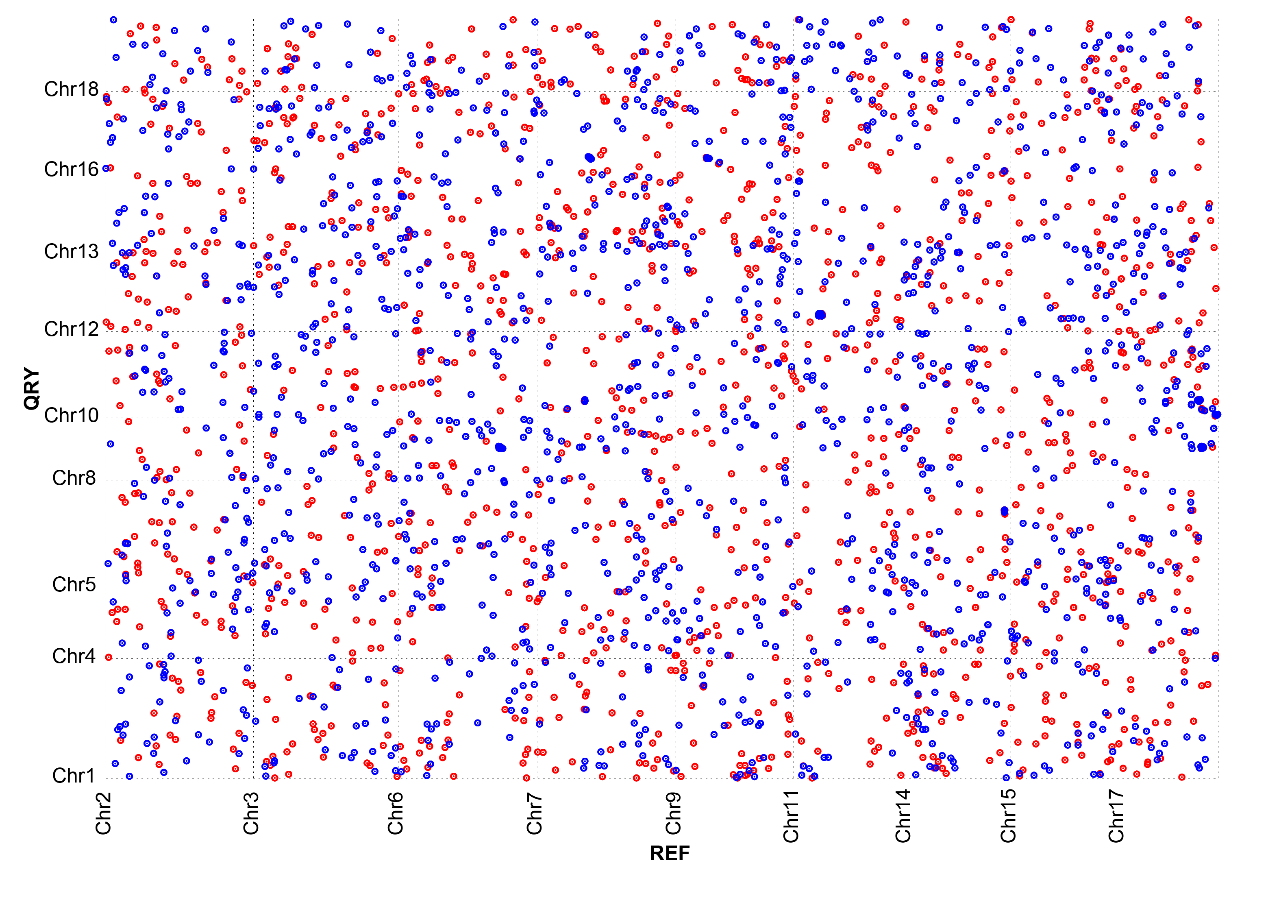


**Figure S8**. Dot plot visualization of collinearity between 18 chromosomes in sub1 at genome level.


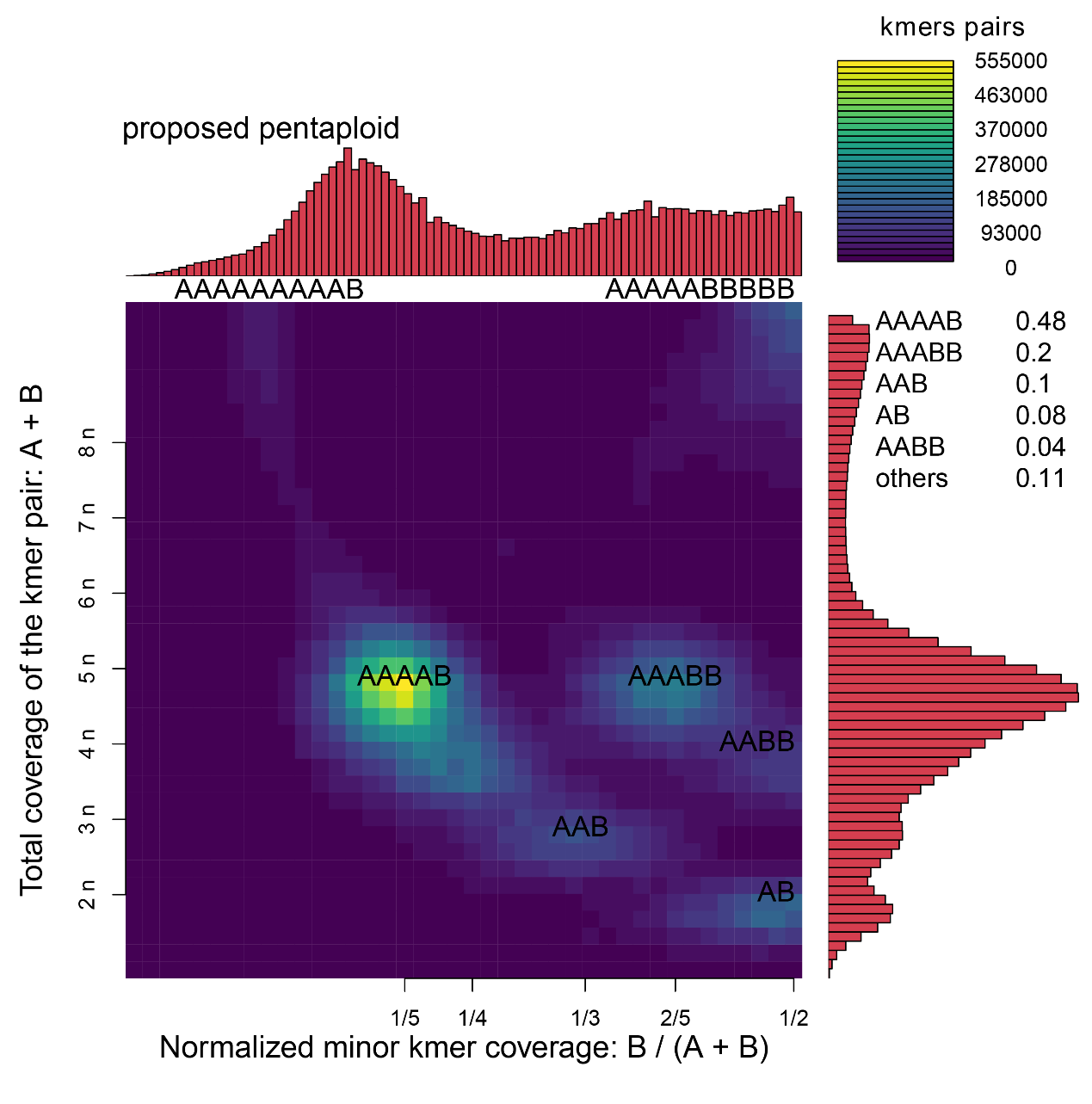


**Figure S9**. Smudgeplot analysis of H. cordata genome.


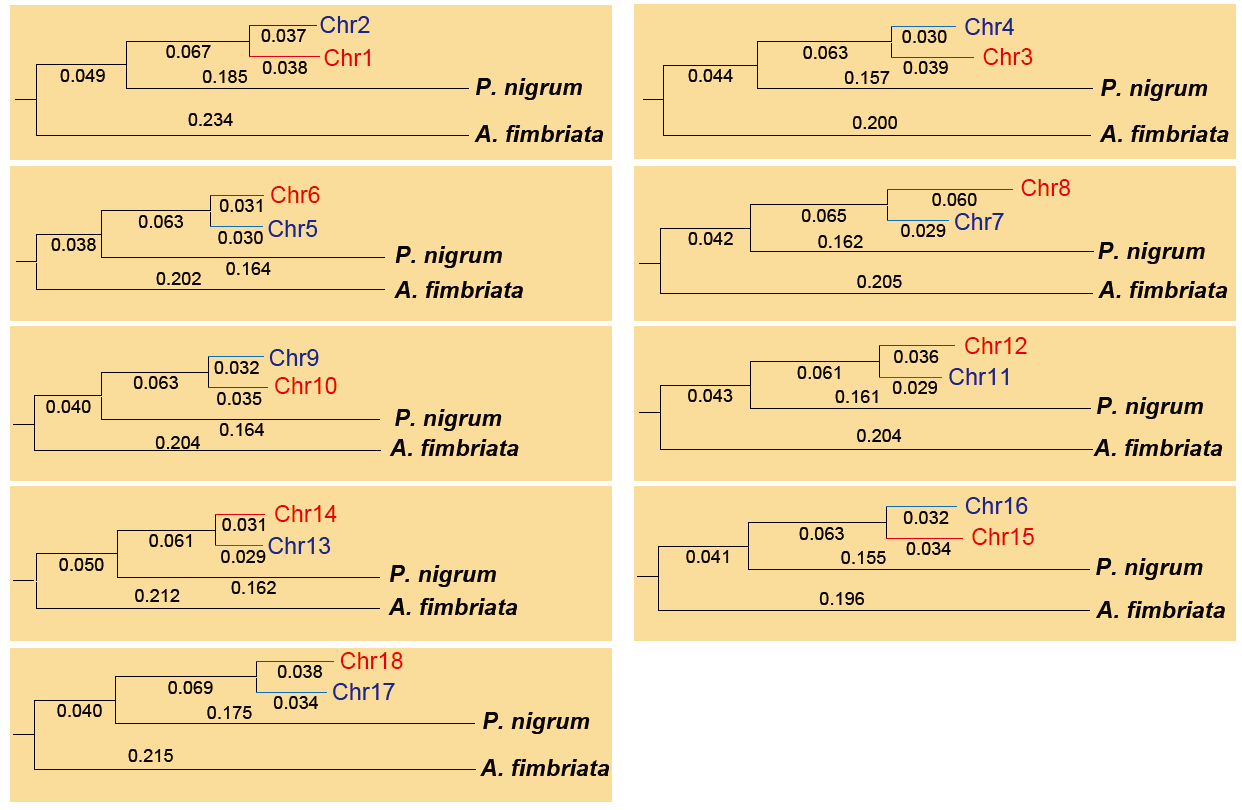


**Figure S10**. The partition and phasing of *H. cordata* subgenomes. Phylogenetic tree based on orthologous genes shows chromosomal relationships between *H. cordata*, *P. nigrum* and *A. fimbriata*. red: A subgenome; blue: B subgenome.


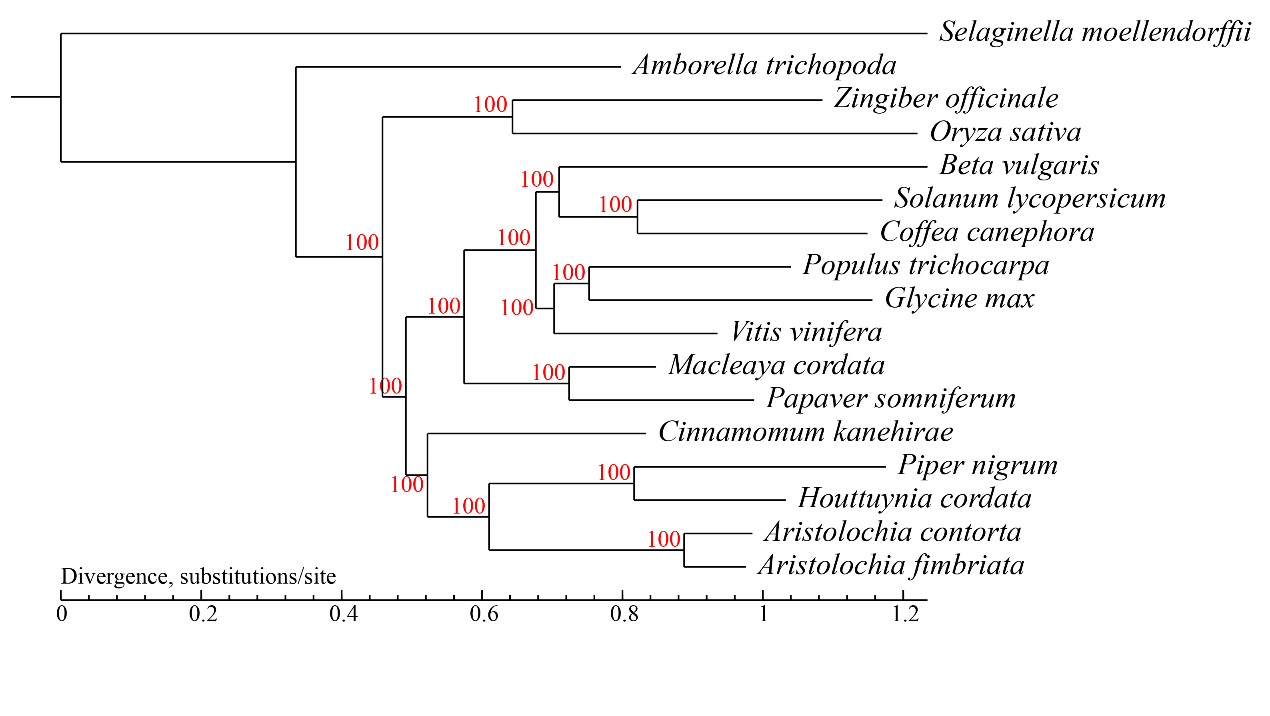


**Figure S11**. Phylogenetic tree with bootstrap support values of each node among 17 plant species.
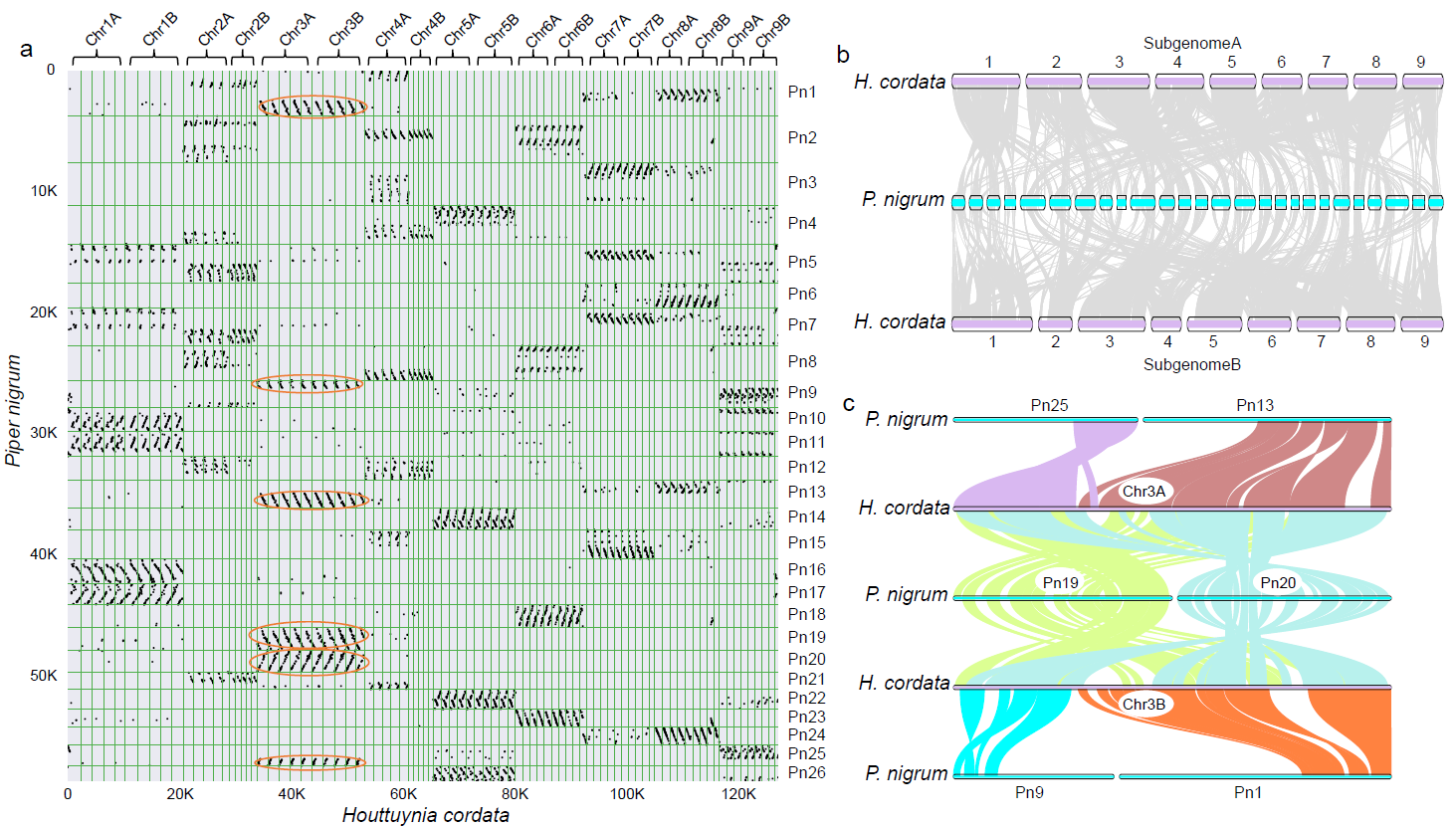


**Figure S12.** (a) Macrosynteny dotplot of *H. cordata* and *P. nigrum* chromosomes. The orange circles indicate the chromosome fusion and duplication events shown in (c). (b) Syntenic relationships among *H. cordata* subgenome and *P. nigrum*. (c) Genomic alignments between Chr3A/3B of *H. cordata* and Pn1, Pn9, Pn13, Pn19, Pn20, Pn25 of *P. nigrum* are shown, corresponds to the orange circle of (a).


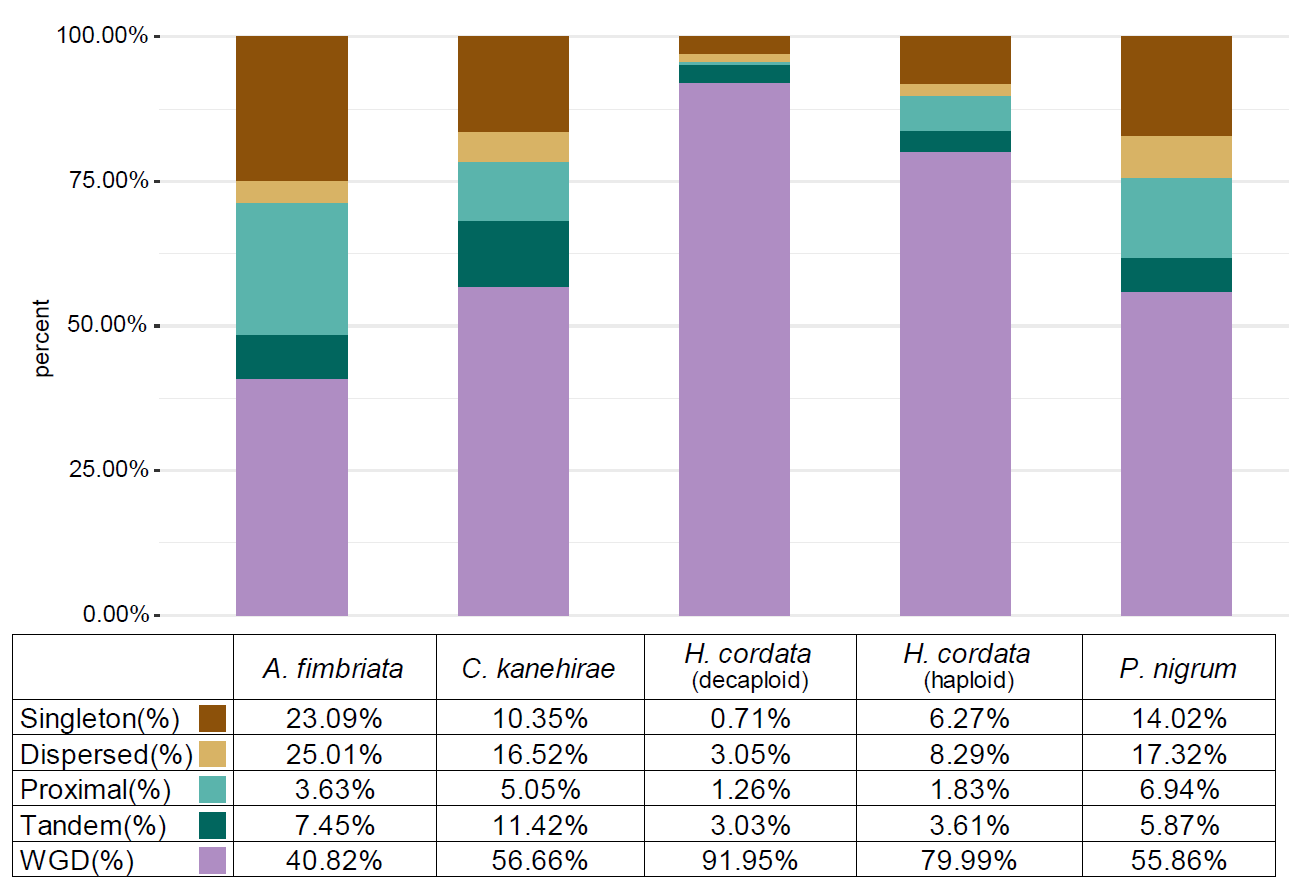


**Figure S13.** The percent of genes from different origins in *H. cordata* and three close-related genomes. The duplication mode were classified into singleton (no duplication), dispersed (duplication type other than WGD/segmental, tandem and proximal), proximal (two duplicated genes are distributed adjacent to each other on chromosomes, with no more than 10 genes spaced but not adjacent), tandem (consecutive repeat) and segmental/whole genome duplications (collinear genes in collinear blocks) using MCScanX (<https://github.com/wyp1125/MCScanX>).


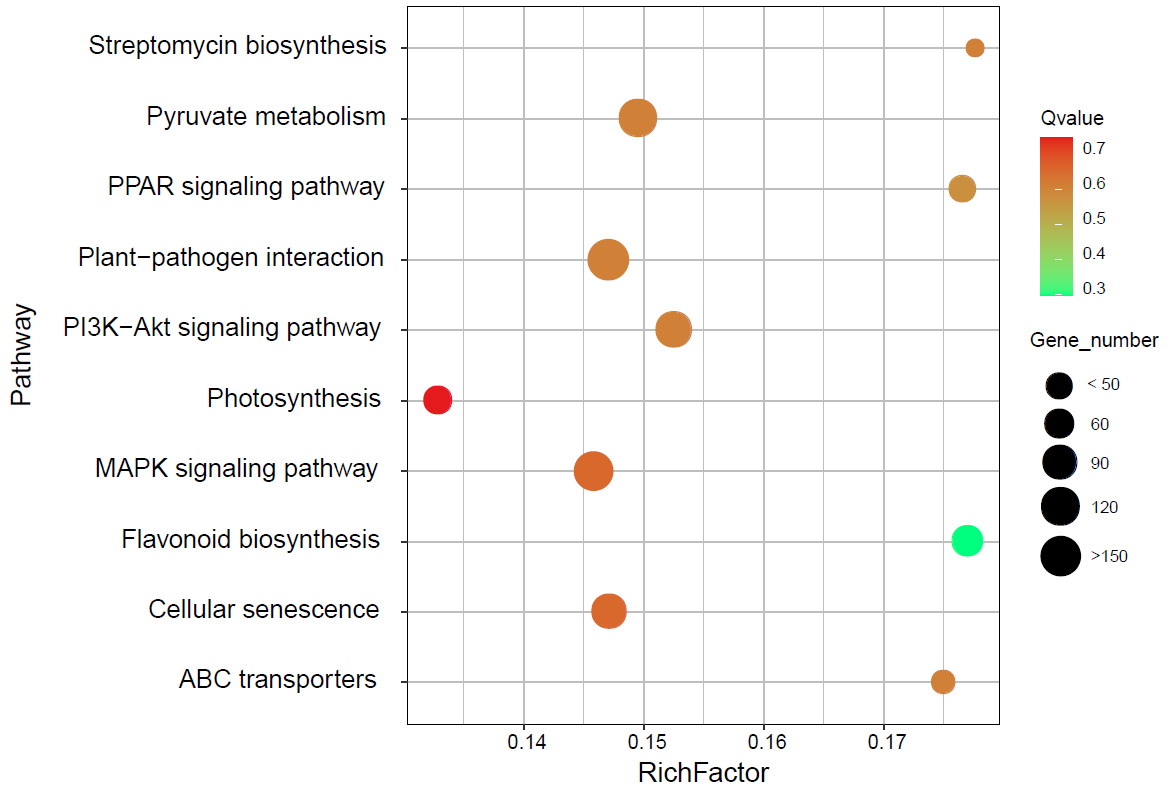


**Figure S14.** KEGG enrichment analysis of *H. cordata* WGD/segmental duplication.


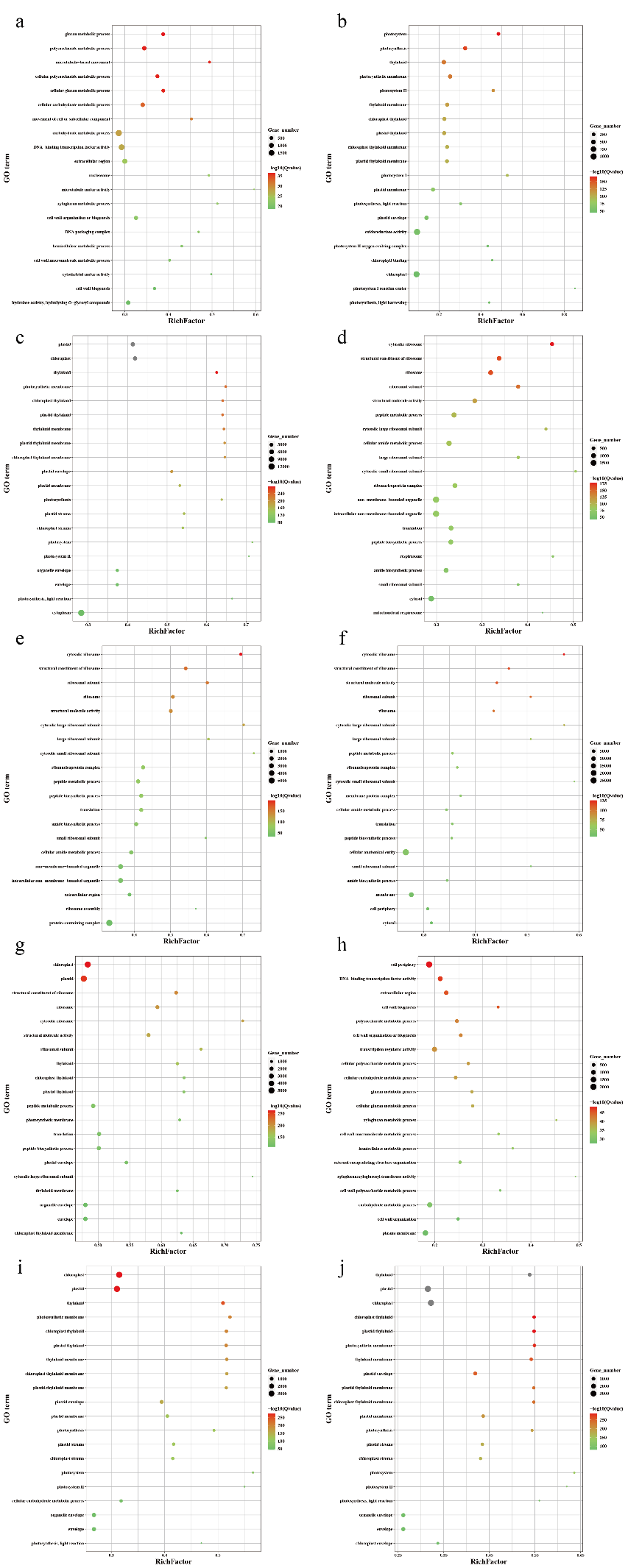


**Supplementary Figure 15.** Comparison of GO enrichment results among different tissue parts. **a** Results of comparison of rhizome and flower. **b** Results of comparison of rhizome and stem. **c** Results of comparison of rhizome and flower. **d** Results of comparison of root and rhizome. **e** Results of comparison of root and flower. **f** Results of comparison of root and stem. **g** Results of comparison of root and leaf. **h** Results of comparison of stem and flower. **i** Results of comparison of stem and leaf. **j** Results of comparison of leaf and flower. The vertical axis is the name of GO term, sorted in ascending order according to Qvalue value from top to small, and the horizontal axis represents RichFactor, the size and color of the dots correspond to the number of differentially expressed genes and the size of QValue, respectively, the larger the number of dots, the redder the color, and the smaller the QValue.


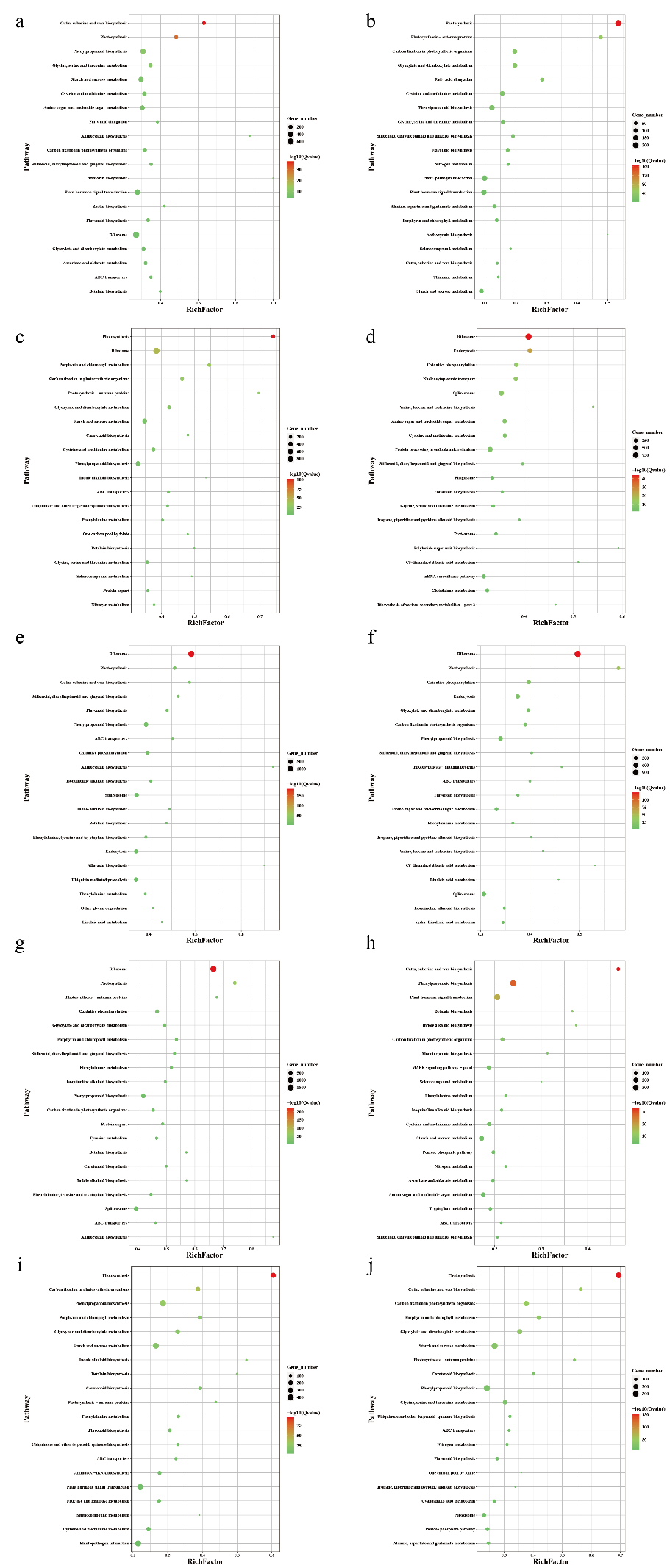


**Supplementary Figure 16.** Comparison of KEGG pathway enrichment results among different tissue parts. **a** Results of comparison of rhizome and flower. **b** Results of comparison of rhizome and stem. **c** Results of comparison of rhizome and flower. **d** Results of comparison of root and rhizome. **e** Results of comparison of root and flower. **f** Results of comparison of root and stem. **g** Results of comparison of root and leaf. **h** Results of comparison of stem and flower. **i** Results of comparison of stem and leaf. **j** Results of comparison of leaf and flower. The vertical axis is the name of KEGG pathway, sorted in ascending order according to Qvalue value from top to small, and the horizontal axis represents RichFactor, the size and color of the dots correspond to the number of differentially expressed genes and the size of QValue, respectively, the larger the number of dots, the redder the color, and the smaller the QValue.


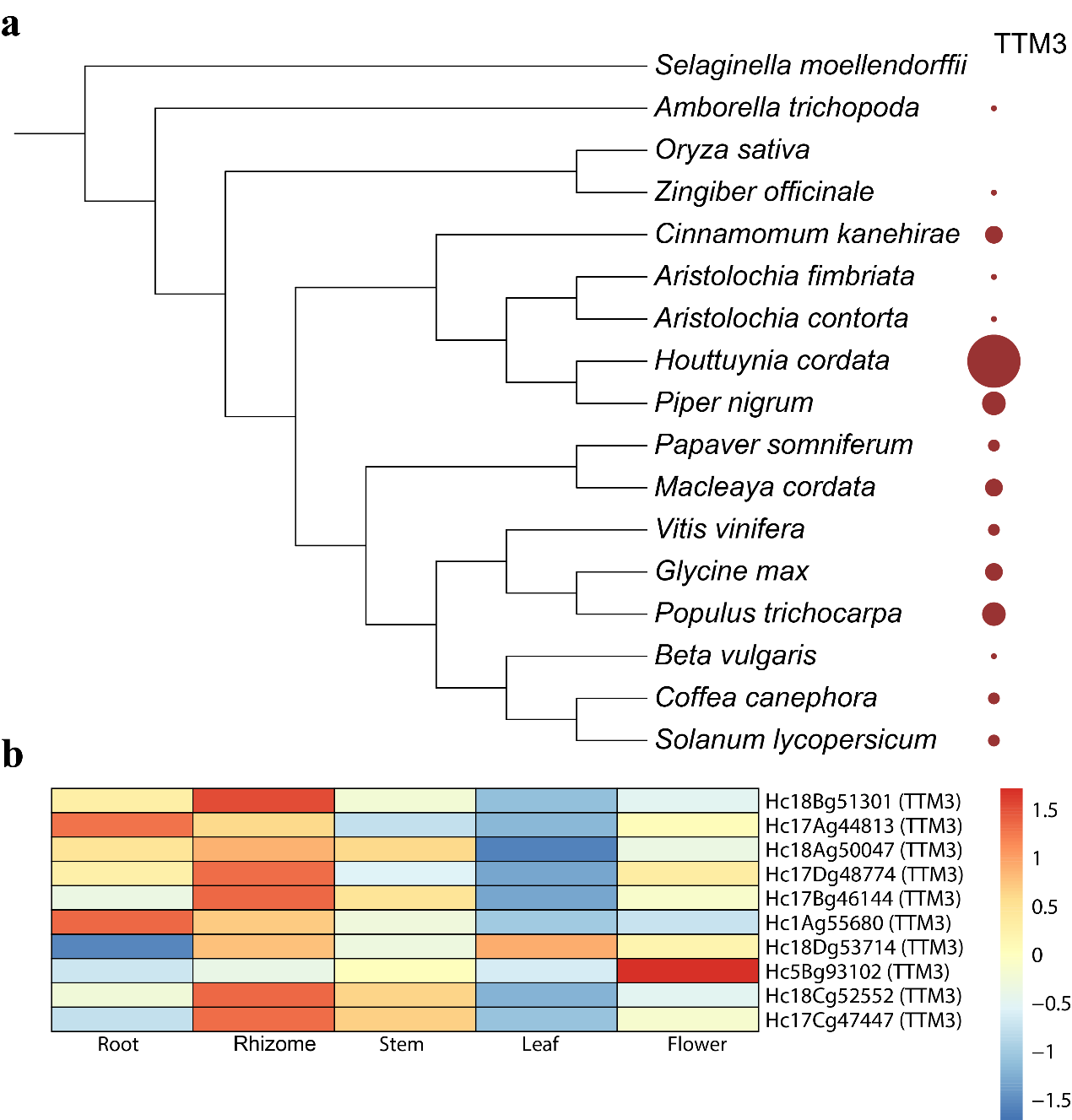


**Figure S17**. (a) Comparison of TTM3 gene amplification among different species. (b) Heat map of TTM3 gene expression in different parts.
