# Supplementary material for: A genome assembly of decaploid Houttuynia cordata provides insights into the evolution of Houttuynia and the biosynthesis of alkaloids
Source: Hortic Res. 2024 Jul 30;11(9):uhae203. doi: 10.1093/hr/uhae203 (PMC11415239; doi:10.1093/hr/uhae203)
Supplement: Web_Material_uhae203 [file web_material_uhae203.zip › Supplementary Tables 1-10.docx]

| **Supplementary Table 1 Sequence Data in *H. cordata*.** | | | |  |
| --- | --- | --- | --- | --- |
| Data type | Platform | Data size (Gb) | Depth(X) | Application |
| HiFi | PacBio Revio | 108.139 | 41.0389 | Genome assembly |
| Hi-C | MGI-SEQ 2000 | 142.74 | 54.1699 | Chromosome construction |

**Supplementary Table 2 The mapping rate of HiFi reads.**

| **data_type** | **Mapping rate (%)** | **Average sequencing depth** | **Coverage (%)** | **Coverage (>= 5X,%)** | **Coverage (>= 10X,%)** | **Coverage(>= 20X,%)** |
| --- | --- | --- | --- | --- | --- | --- |
| **HiFi** | **99.77** | **40.43** | **99.97** | **99.51** | **98.99** | **97.6** |

**Supplementary Table 3 BUSCO evaluation of *H. cordata* genome assembly and annotation.**

|  | Assembly | | Annotation | |
| --- | --- | --- | --- | --- |
|  | Proteins | Percentages(%) | Proteins | Percentages(%) |
| Complete BUSCOs | 1,594 | 98.8 | 1,605 | 99.4 |
| Complete and single-copy BUSCOs | 8 | 0.5 | 13 | 0.8 |
| Complete and duplicated BUSCOs | 1,586 | 98.3 | 1,592 | 98.6 |
| Fragmented BUSCOs | 11 | 0.7 | 3 | 0.2 |
| Missing BUSCOs | 9 | 0.6 | 6 | 0.4 |
| Total BUSCO groups searched | 1,614 | 100 | 1,614 | 100 |

**Supplementary Table 4 Repeat classification in *H. cordata*.**

| **Repeat Class Description** |  | **length(bp)** | **percent(%)** |
| --- | --- | --- | --- |
| Class I: Retrotransposon | LINE | 75,835,985 | 2.88 |
|  | SINE | 49,583 | 0 |
|  | LTR | 851,900,498 | 32.33 |
| Class II: DNA transposon | DNA | 530,754,725 | 20.14 |
| Other |  | 55,462,826 | 2.1 |
| Total TE |  | 1,462,909,981 | 55.52 |

**Supplementary Table 5 Summary of predicted non-coding RNAs in *H. cordata*.**

| Type | Total | Average Length (bp) | Total Length (bp) | % of Genome |
| --- | --- | --- | --- | --- |
| miRNA | 504 | 127 | 63,917 | 0.00 |
| tRNA | 4,744 | 78 | 367,696 | 0.01 |
| rRNA | 2,386 | 2107 | 5,026,462 | 0.19 |
| snRNA | 893 | 128 | 114,746 | 0.00 |

**Supplementary Table 6 Functional annotation of predicted genes in *H. cordata*.**

| Database | Number | Percent |
| --- | --- | --- |
| InterPro | 110,174 | 79.21% |
| GO | 116,707 | 83.91% |
| KEGG | 135,172 | 97.19% |
| Swissprot | 107,195 | 77.07% |
| TrEMBL | 135,593 | 97.49% |
| NR | 135,739 | 97.59% |
| Annotated | 136,006 | 97.78% |

**Supplementary Table 7 Pseudomolecule length of *H. cordata* two subgenomes**

| **OldName** | **NewName** | **Length (bp)** | **OldName** | **NewName** | **Length (bp)** |
| --- | --- | --- | --- | --- | --- |
| Chr1_sub1 | Chr1A1 | 39,589,471 | Chr2_sub1 | Chr1B1 | 36,660,000 |
| Chr1_sub2 | Chr1A2 | 36,760,226 | Chr2_sub2 | Chr1B2 | 35,873,154 |
| Chr1_sub3 | Chr1A3 | 36,233,277 | Chr2_sub3 | Chr1B3 | 35,343,020 |
| Chr1_sub4 | Chr1A4 | 35,567,232 | Chr2_sub4 | Chr1B4 | 31,902,904 |
| Chr1_sub5 | Chr1A5 | 32,055,549 | Chr2_sub5 | Chr1B5 | 35,088,739 |
| Chr3_sub1 | Chr2A1 | 35,841,941 | Chr4_sub1 | Chr2B1 | 23,685,105 |
| Chr3_sub2 | Chr2A2 | 35,379,421 | Chr4_sub2 | Chr2B2 | 23,378,102 |
| Chr3_sub3 | Chr2A3 | 34,472,585 | Chr4_sub3 | Chr2B3 | 23,317,395 |
| Chr3_sub4 | Chr2A4 | 32,536,425 | Chr4_sub4 | Chr2B4 | 22,396,540 |
| Chr3_sub5 | Chr2A5 | 28,341,155 | Chr4_sub5 | Chr2B5 | 22,118,863 |
| Chr6_sub1 | Chr3A1 | 34,438,007 | Chr5_sub1 | Chr3B1 | 35,374,509 |
| Chr6_sub2 | Chr3A2 | 34,184,365 | Chr5_sub2 | Chr3B2 | 35,251,249 |
| Chr6_sub3 | Chr3A3 | 33,374,122 | Chr5_sub3 | Chr3B3 | 34,913,231 |
| Chr6_sub4 | Chr3A4 | 30,742,670 | Chr5_sub4 | Chr3B4 | 34,253,303 |
| Chr6_sub5 | Chr3A5 | 27,008,281 | Chr5_sub5 | Chr3B5 | 32,647,851 |
| Chr8_sub1 | Chr4A1 | 20,681,036 | Chr7_sub1 | Chr4B1 | 34,455,768 |
| Chr8_sub2 | Chr4A2 | 20,381,373 | Chr7_sub2 | Chr4B2 | 32,578,785 |
| Chr8_sub3 | Chr4A3 | 19,421,149 | Chr7_sub3 | Chr4B3 | 32,348,785 |
| Chr8_sub4 | Chr4A4 | 18,604,663 | Chr7_sub4 | Chr4B4 | 31,512,533 |
| Chr8_sub5 | Chr4A5 | 17,971,310 | Chr7_sub5 | Chr4B5 | 31,076,003 |
| Chr10_sub1 | Chr5A1 | 28,572,027 | Chr9_sub1 | Chr5B1 | 29,199,750 |
| Chr10_sub2 | Chr5A2 | 26,636,080 | Chr9_sub2 | Chr5B2 | 27,485,493 |
| Chr10_sub3 | Chr5A3 | 26,271,290 | Chr9_sub3 | Chr5B3 | 26,945,135 |
| Chr10_sub4 | Chr5A4 | 25,222,857 | Chr9_sub4 | Chr5B4 | 25,388,961 |
| Chr10_sub5 | Chr5A5 | 24,576,516 | Chr9_sub5 | Chr5B5 | 25,115,429 |
| Chr12_sub1 | Chr6A1 | 25,734,424 | Chr11_sub1 | Chr6B1 | 27,829,033 |
| Chr12_sub2 | Chr6A2 | 24,792,996 | Chr11_sub2 | Chr6B2 | 27,360,559 |
| Chr12_sub3 | Chr6A3 | 24,698,245 | Chr11_sub3 | Chr6B3 | 27,042,361 |
| Chr12_sub4 | Chr6A4 | 24,610,528 | Chr11_sub4 | Chr6B4 | 25,924,646 |
| Chr12_sub5 | Chr6A5 | 23,749,693 | Chr11_sub5 | Chr6B5 | 25,899,305 |
| Chr14_sub1 | Chr7A1 | 26,086,927 | Chr13_sub1 | Chr7B1 | 27,206,879 |
| Chr14_sub2 | Chr7A2 | 24,304,215 | Chr13_sub2 | Chr7B2 | 24,476,086 |
| Chr14_sub3 | Chr7A3 | 24,262,133 | Chr13_sub3 | Chr7B3 | 24,346,403 |
| Chr14_sub4 | Chr7A4 | 24,183,112 | Chr13_sub4 | Chr7B4 | 22,877,233 |
| Chr14_sub5 | Chr7A5 | 20,521,950 | Chr13_sub5 | Chr7B5 | 22,163,702 |
| Chr15_sub1 | Chr8A1 | 26,779,062 | Chr16_sub1 | Chr8B1 | 26,518,778 |
| Chr15_sub2 | Chr8A2 | 26,300,312 | Chr16_sub2 | Chr8B2 | 25,422,575 |
| Chr15_sub3 | Chr8A3 | 26,101,091 | Chr16_sub3 | Chr8B3 | 25,137,492 |
| Chr15_sub4 | Chr8A4 | 24,843,410 | Chr16_sub5 | Chr8B4 | 18,760,037 |
|  |  |  | Chr16_sub4 | Chr8B5 | 24,119,181 |
| Chr18_sub1 | Chr9A1 | 23,997,983 | Chr17_sub1 | Chr9B1 | 24,644,447 |
| Chr18_sub2 | Chr9A2 | 22,407,281 | Chr17_sub2 | Chr9B2 | 24,197,824 |
| Chr18_sub3 | Chr9A3 | 18,605,440 | Chr17_sub3 | Chr9B3 | 23,983,144 |
| Chr18_sub4 | Chr9A4 | 17,417,277 | Chr17_sub4 | Chr9B4 | 22,178,626 |
| Chr18_sub5 | Chr9A5 | 11,340,767 |  |  |  |
| Total size |  | 1,175,599,874 |  |  | 1,228,398,918 |

**Supplementary Table 8 KEGG enrichment of *H. cordata* expansion gene families**

| **pathway** | **pathway id** | **gene Number** | **p-value** | **FDR** |
| --- | --- | --- | --- | --- |
| Phenylpropanoid biosynthesis | ko00940 | 287 | 1.90E-17 | 1.54E-16 |
| Isoquinoline alkaloid biosynthesis | ko00950 | 74 | 1.21E-08 | 4.26E-08 |
| Flavonoid biosynthesis | ko00941 | 92 | 2.90E-08 | 9.94E-08 |
| Diterpenoid biosynthesis | ko00904 | 35 | 1.55E-05 | 4.14E-05 |
| Tropane, piperidine and pyridine alkaloid biosynthesis | ko00960 | 34 | 9.73E-03 | 1.86E-02 |
| Betalain biosynthesis | ko00965 | 65 | 1.22E-26 | 2.12E-25 |
| Indole alkaloid biosynthesis | ko00901 | 51 | 1.20E-18 | 1.04E-17 |
| Novobiocin biosynthesis | ko00401 | 15 | 9.33E-08 | 3.11E-07 |
| Stilbenoid, diarylheptanoid and gingerol biosynthesis | ko00945 | 70 | 1.36E-05 | 3.67E-05 |
| Neomycin, kanamycin and gentamicin biosynthesis | ko00524 | 10 | 4.40E-02 | 7.87E-02 |
| Monobactam biosynthesis | ko00261 | 16 | 2.32E-01 | 3.92E-01 |
| Streptomycin biosynthesis | ko00521 | 10 | 9.63E-01 | 1.00E+00 |

**Supplementary Table 9**. Number of gene pairs and homologous blocks within four Magnoliaceae genomes.

| Species | Blocks | Genepairs |
| --- | --- | --- |
| *H. cordata-H. cordata* | 3,753 | 199,329 |
| *P. nigrum*-*P. nigrum* | 586 | 12,281 |
| *A. fimbriata*-*A. fimbriata* | 48 | 237 |
| *C. kanehirae*-*C. kanehirae* | 312 | 4,397 |
| *H. cordata*-*P. nigrum* | 8,431 | 139,643 |

**Supplementary Table 10. Sequence expression and annotation of genes in each group.** The first column is the sequences number, the second column is the annotated gene name, and columns 3-7 are the average expression values of the sequence in roots, rhizomes, stems, leaves and flowers, respectively.

| **gene_id** | **group** | **YXC-G** | **YXC-GJ** | **YXC-J** | **YXC-Y** | **YXC-H** |
| --- | --- | --- | --- | --- | --- | --- |
| Hc6Dg107326 | 4OMT | 1.46 | 2.58 | 8.19 | 0.32 | 2.86 |
| Hc6Eg108965 | 4OMT | 0.44 | 3 | 4.32 | 0.08 | 3.07 |
| Hc13Cg23524 | 4OMT | 1.76 | 3.46 | 2.56 | 1.35 | 2.34 |
| Hc13Cg23525 | 4OMT | 0.16 | 0.16 | 0.26 | 0 | 6.8 |
| Hc6Ag101386 | 4OMT | 0.79 | 0.85 | 0.03 | 0.18 | 0 |
| Hc5Ag90717 | 4OMT | 0.27 | 0.37 | 1.68 | 0.14 | 1.75 |
| Hc16Bg40868 | 4OMT | 0 | 0 | 0.09 | 0 | 0 |
| Hc6Cg105458 | 4OMT | 8.92 | 18.31 | 18.81 | 0.61 | 6.49 |
| Hc13Bg22277 | 4OMT | 0.82 | 1.75 | 2.35 | 0.95 | 1.7 |
| Hc13Bg22278 | 4OMT | 0 | 0.03 | 0 | 0 | 1.06 |
| Hc5Bg92679 | 4OMT | 0.26 | 0.23 | 0.92 | 0.12 | 0.97 |
| Hc13Ag21003 | 4OMT | 2.36 | 2.93 | 3.09 | 1.14 | 1.23 |
| Hc13Ag21004 | 4OMT | 0.53 | 0.11 | 0.31 | 0 | 9.62 |
| Hc13Dg24798 | 4OMT | 3.2 | 3.3 | 5.05 | 1.59 | 2.06 |
| Hc5Cg94705 | 4OMT | 0.47 | 0.12 | 0.32 | 0.05 | 1.03 |
| Hc6Bg103436 | 4OMT | 0.22 | 0.23 | 0.95 | 0 | 0.32 |
| Hc12Eg20276 | 4OMT | 0.45 | 4.13 | 5.14 | 0 | 0.27 |
| Hc16Ag39563 | 4OMT | 0.12 | 0.17 | 0.86 | 0.17 | 12.71 |
| Hc16Ag39564 | 4OMT | 1.06 | 0.9 | 1.35 | 0.88 | 1.13 |
| Hc16Dg43064 | 6OMT | 1.9 | 0 | 0.16 | 0 | 0.96 |
| Hc16Dg43065 | 6OMT | 11.72 | 0.05 | 0.78 | 0.01 | 1.19 |
| Hc12Dg18134 | 6OMT | 25.77 | 0.91 | 0.63 | 0 | 0.47 |
| Hc12Cg16877 | 6OMT | 31.91 | 0.68 | 0.61 | 0.09 | 0.37 |
| Hc16Bg41148 | 6OMT | 0.76 | 0 | 0.22 | 0 | 2.96 |
| Hc18Ag50871 | 6OMT | 0.35 | 0.22 | 0.11 | 0.04 | 0.6 |
| Hc15Ag34237 | 6OMT | 0.11 | 0.17 | 0.1 | 0 | 3.12 |
| Hc18Dg54542 | 6OMT | 0.21 | 0.17 | 0.16 | 0 | 3.24 |
| Hc18Bg52129 | 6OMT | 0.88 | 0.11 | 0.03 | 0.05 | 0.43 |
| Hc16Cg41742 | 6OMT | 3.85 | 0 | 0.13 | 0.05 | 0.24 |
| Hc13Eg25777 | 6OMT | 0.3 | 0.02 | 0.04 | 0.02 | 0.93 |
| Hc12Eg19435 | 6OMT | 28.37 | 1.01 | 0.58 | 0 | 1 |
| Hc12Eg20543 | 6OMT | 0 | 0 | 0 | 0 | 0.08 |
| Hc15Dg38398 | 6OMT | 0.26 | 0.07 | 0.05 | 0 | 0.52 |
| Hc18Cg53394 | 6OMT | 0.98 | 0.32 | 0.13 | 0.1 | 0.73 |
| Hc12Ag15373 | 6OMT | 68.71 | 2.19 | 1.18 | 0 | 0.81 |
| Hc16Ag38972 | 6OMT | 9.27 | 0 | 0.08 | 0.06 | 0.35 |
| Hc15Bg34877 | 6OMT | 0.76 | 0 | 0.23 | 0 | 0.36 |
| Hc15Bg35622 | 6OMT | 0 | 0.02 | 0 | 0 | 0.06 |
| Hc15Cg36253 | 6OMT | 4.48 | 0 | 0.25 | 0.09 | 0.66 |
| Hc15Cg37009 | 6OMT | 0.32 | 0.15 | 0 | 0.16 | 0.24 |
| Hc15Cg37010 | 6OMT | 0 | 0 | 0 | 0 | 0.19 |
| Hc18Eg55429 | 6OMT | 0.84 | 0.13 | 0 | 0 | 1.24 |
| Hc12Bg15610 | 6OMT | 3.67 | 0.06 | 0.1 | 0 | 0.16 |
| unanchor174g131894 | 6OMT | 10.24 | 0 | 0.34 | 0.04 | 0.51 |
| unanchor370g136386 | 6OMT | 0.83 | 0 | 0.04 | 0 | 0.17 |
| unanchor374g136399 | 6OMT | 0.63 | 0.05 | 0.02 | 0.18 | 0.47 |
| Hc12Dg19184 | BBE | 1.3 | 3.1 | 0.8 | 0.29 | 0.13 |
| Hc4Dg88227 | BBE | 0.35 | 0 | 0.06 | 8.38 | 2.96 |
| Hc12Cg17877 | BBE | 0.51 | 0.55 | 0.25 | 0 | 0.08 |
| Hc8Eg121339 | BBE | 2.36 | 0.09 | 0.02 | 0.13 | 0.48 |
| Hc8Eg121341 | BBE | 0 | 0.01 | 1.17 | 0 | 0 |
| Hc7Eg116151 | BBE | 0.06 | 0.05 | 0.02 | 0.1 | 0.05 |
| Hc7Eg116152 | BBE | 2.21 | 7.25 | 2.29 | 0 | 0.71 |
| Hc7Eg116154 | BBE | 0.12 | 0 | 2.74 | 0.24 | 0.33 |
| Hc7Eg116155 | BBE | 0 | 0 | 0 | 0.02 | 0 |
| Hc7Eg116408 | BBE | 0.47 | 0.05 | 0 | 0 | 0.04 |
| Hc8Cg119556 | BBE | 12.54 | 6.32 | 1.53 | 1.98 | 3.91 |
| Hc8Cg119557 | BBE | 0 | 0.49 | 1.74 | 0.08 | 0.13 |
| Hc7Ag109820 | BBE | 0.04 | 0.09 | 0.12 | 0.54 | 0.26 |
| Hc7Ag109821 | BBE | 5.49 | 5.9 | 6.26 | 0.22 | 0.97 |
| Hc7Ag110063 | BBE | 1.66 | 0.24 | 0.41 | 0.48 | 3.59 |
| Hc11Ag08814 | BBE | 0.52 | 0 | 0 | 0.07 | 0.63 |
| Hc4Cg87277 | BBE | 0.9 | 0 | 0 | 0.97 | 0.86 |
| Hc8Ag117776 | BBE | 11.35 | 37.83 | 11.08 | 1.11 | 7.41 |
| Hc8Ag117779 | BBE | 2.09 | 0 | 0 | 0.02 | 0 |
| Hc8Ag117781 | BBE | 0 | 0.01 | 1.17 | 0 | 0 |
| Hc8Dg120455 | BBE | 0 | 0.14 | 0.95 | 0.13 | 0.05 |
| Hc4Ag85290 | BBE | 0.11 | 0 | 0 | 4.97 | 1.5 |
| Hc11Cg11422 | BBE | 0 | 0 | 0 | 0 | 0.2 |
| Hc4Eg89208 | BBE | 1.23 | 0.16 | 1.38 | 9.79 | 8.97 |
| Hc11Dg12730 | BBE | 2.86 | 2.47 | 1.66 | 2.65 | 2.09 |
| Hc11Dg12731 | BBE | 0.21 | 0.01 | 0.04 | 0.41 | 0.67 |
| Hc7Dg114568 | BBE | 0.06 | 0.29 | 0.22 | 1.21 | 0.49 |
| Hc7Dg114808 | BBE | 2.31 | 0.8 | 0.1 | 0.03 | 0.06 |
| Hc7Bg111412 | BBE | 0 | 0.11 | 0.54 | 0 | 0.13 |
| Hc7Bg111654 | BBE | 0.22 | 0 | 0.03 | 0.03 | 0.08 |
| Hc7Cg112990 | BBE | 0.6 | 0.4 | 0.38 | 1.05 | 0.46 |
| Hc7Cg112991 | BBE | 14.55 | 40.98 | 20.29 | 0.86 | 3.99 |
| Hc7Cg112992 | BBE | 0.11 | 0.56 | 1.95 | 0.06 | 2.34 |
| Hc7Cg112993 | BBE | 0 | 0 | 0 | 0 | 0.02 |
| Hc7Cg113245 | BBE | 1.29 | 0.09 | 0.21 | 0.21 | 2.54 |
| Hc4Bg86283 | BBE | 0.49 | 0 | 0.34 | 1.94 | 2.27 |
| Hc8Bg118659 | BBE | 0 | 0.17 | 1.02 | 0.02 | 0.08 |
| Hc11Bg10123 | BBE | 0 | 0.17 | 0.06 | 0.43 | 0.39 |
| Hc12Bg16623 | BBE | 1.73 | 0.45 | 0.01 | 0 | 0.04 |
| Hc5Cg95279 | CFS | 1.14 | 0.28 | 0.88 | 0.34 | 1.19 |
| Hc3Ag77873 | CNMT | 3.42 | 0.03 | 0.05 | 0.12 | 0.39 |
| Hc3Cg81636 | CNMT | 1.24 | 0 | 0.02 | 0.68 | 1.47 |
| Hc3Cg81635 | CNMT | 4.24 | 0 | 0.11 | 0.09 | 0.22 |
| Hc4Dg88405 | CNMT | 2.48 | 1.04 | 4.01 | 6.37 | 5.39 |
| Hc7Eg116508 | CNMT | 2.21 | 0.39 | 0.09 | 0.62 | 3.55 |
| Hc7Eg116509 | CNMT | 3.69 | 0.31 | 0.43 | 0.16 | 3.55 |
| Hc3Eg84474 | CNMT | 4.71 | 0.04 | 0.02 | 0.08 | 0.32 |
| Hc7Ag110210 | CNMT | 2.56 | 2.79 | 1.82 | 1.88 | 10.08 |
| Hc7Ag110221 | CNMT | 11.23 | 4.99 | 5.07 | 5.73 | 40.03 |
| Hc4Cg87444 | CNMT | 3.94 | 0 | 0 | 0 | 0 |
| Hc3Bg79678 | CNMT | 3.48 | 0 | 0.21 | 0.08 | 0.41 |
| Hc3Bg79677 | CNMT | 0 | 0 | 0 | 0 | 1.25 |
| Hc4Ag85460 | CNMT | 3.37 | 2.37 | 3.92 | 7.92 | 4.9 |
| Hc4Eg89375 | CNMT | 3.92 | 3.62 | 5.82 | 10.35 | 7.21 |
| Hc7Dg114955 | CNMT | 17.67 | 1.61 | 2.06 | 1.51 | 20.66 |
| Hc7Bg111800 | CNMT | 1.25 | 0.43 | 1.37 | 1.67 | 8.94 |
| Hc7Bg111801 | CNMT | 4.29 | 0.32 | 0.32 | 0.21 | 2.4 |
| Hc7Cg113364 | CNMT | 7.51 | 0.63 | 0.56 | 0.43 | 4.9 |
| Hc3Dg83178 | CNMT | 4.09 | 0.02 | 0 | 0 | 0.14 |
| Hc4Bg86435 | CNMT | 8.86 | 9.45 | 10.04 | 22.47 | 13.84 |
| unanchor1g130447 | CNMT | 0.12 | 0 | 0 | 0 | 0 |
| unanchor2g133293 | CNMT | 0.24 | 0 | 0.03 | 0 | 0.03 |
| unanchor3g135428 | CNMT | 0.69 | 0 | 0.1 | 0 | 0.07 |
| unanchor4g137373 | CNMT | 0.8 | 0 | 0.04 | 0.06 | 0.09 |
| unanchor5g139230 | CNMT | 2.08 | 0 | 0.07 | 0 | 0 |
| Hc13Cg23412 | DDC | 3 | 1.21 | 10.64 | 2.28 | 0.7 |
| Hc16Dg43097 | DDC | 20.65 | 9.17 | 6.65 | 3.15 | 2.77 |
| Hc6Ag100315 | DDC | 0 | 0 | 0.02 | 0 | 0 |
| Hc3Ag77412 | DDC | 15.35 | 2.48 | 0.59 | 0.13 | 0.11 |
| Hc2Eg75014 | DDC | 0.09 | 0.02 | 0.04 | 0.02 | 0.56 |
| Hc14Dg31957 | DDC | 0.22 | 0.01 | 0.01 | 0.02 | 0 |
| Hc14Dg31414 | DDC | 1.18 | 3.31 | 3.06 | 6.65 | 2.95 |
| Hc3Cg81056 | DDC | 10.9 | 2.86 | 5.76 | 1.42 | 1 |
| Hc3Cg80827 | DDC | 0 | 0 | 0.01 | 0.01 | 0 |
| Hc3Cg80476 | DDC | 57.31 | 39.29 | 48.05 | 12.11 | 11.37 |
| Hc3Cg80475 | DDC | 29.48 | 28.79 | 50.51 | 4.83 | 13.58 |
| Hc4Dg88437 | DDC | 19.74 | 9.36 | 18 | 1.49 | 5.46 |
| Hc4Dg88587 | DDC | 56.04 | 27.11 | 58.14 | 14.05 | 7.7 |
| Hc4Dg88940 | DDC | 0.29 | 1.32 | 1.93 | 0.69 | 0.84 |
| Hc4Dg88941 | DDC | 22.83 | 25.49 | 39.02 | 4.53 | 10.65 |
| Hc4Dg88942 | DDC | 26.22 | 14.06 | 19.01 | 2.55 | 9.58 |
| Hc8Eg121202 | DDC | 57.72 | 25.98 | 16.73 | 9.1 | 26.73 |
| Hc8Eg121580 | DDC | 0.15 | 0.43 | 0.49 | 0.25 | 0.45 |
| Hc2Ag66534 | DDC | 0.07 | 0 | 0 | 0.04 | 0.32 |
| Hc7Eg116024 | DDC | 77.94 | 44.51 | 8.51 | 0.7 | 8.66 |
| Hc7Eg116510 | DDC | 0 | 0 | 0.02 | 0 | 0 |
| Hc7Eg116518 | DDC | 27.27 | 4.54 | 8.6 | 0.81 | 1.2 |
| Hc2Dg73064 | DDC | 0 | 0 | 0.07 | 0.07 | 0.21 |
| Hc8Cg119404 | DDC | 82.73 | 32.35 | 16 | 10.86 | 19.9 |
| Hc8Cg119807 | DDC | 0.01 | 0 | 0 | 0 | 0.06 |
| Hc8Cg119808 | DDC | 0.01 | 0 | 0 | 0 | 0.04 |
| Hc8Cg119809 | DDC | 0.5 | 3.37 | 11.34 | 0.32 | 3.45 |
| Hc16Bg40455 | DDC | 9.23 | 4.64 | 2.34 | 0.39 | 0.51 |
| Hc3Eg84014 | DDC | 14.39 | 2.14 | 1.52 | 0.38 | 0.52 |
| Hc7Ag109692 | DDC | 139.41 | 97.58 | 13.15 | 1.84 | 15.52 |
| Hc7Ag110081 | DDC | 47.32 | 30.06 | 69.08 | 21.09 | 12.6 |
| Hc11Ag07817 | DDC | 5.32 | 2.44 | 0.33 | 0.02 | 0.06 |
| Hc4Cg87482 | DDC | 18.32 | 17.03 | 28.86 | 3.86 | 7.92 |
| Hc4Cg87624 | DDC | 70.64 | 31.49 | 22.09 | 5.25 | 4.13 |
| Hc4Cg87976 | DDC | 22.4 | 17.11 | 27.29 | 4.18 | 5.65 |
| Hc4Cg87977 | DDC | 25.44 | 27.42 | 48.65 | 7.26 | 7.12 |
| Hc4Cg87978 | DDC | 59.18 | 54.07 | 57.3 | 9.96 | 19.65 |
| Hc13Bg22163 | DDC | 16.15 | 15.47 | 36.87 | 14.69 | 3.83 |
| Hc16Cg42518 | DDC | 0.02 | 0.01 | 0 | 0 | 0 |
| Hc8Ag117644 | DDC | 57.4 | 47.74 | 32.66 | 8.65 | 22.39 |
| Hc8Dg120325 | DDC | 59.06 | 26.94 | 20.72 | 15.09 | 43.11 |
| Hc14Bg29380 | DDC | 0.02 | 0 | 0 | 0 | 0.03 |
| Hc14Bg28844 | DDC | 0.6 | 0.56 | 0.69 | 2.92 | 0.78 |
| Hc13Dg24688 | DDC | 6.85 | 3.84 | 42.57 | 14.46 | 4.71 |
| Hc3Bg79241 | DDC | 26.92 | 6.35 | 4.86 | 0.71 | 1.11 |
| Hc3Bg79003 | DDC | 0 | 0 | 0 | 0.14 | 0 |
| Hc3Bg78657 | DDC | 27.12 | 12.48 | 28.5 | 7.38 | 4.69 |
| Hc3Bg78656 | DDC | 10.08 | 3.16 | 7.53 | 1.28 | 1.17 |
| Hc4Ag85494 | DDC | 23.21 | 7.87 | 7.31 | 2.49 | 4.82 |
| Hc4Ag85644 | DDC | 14.4 | 2.76 | 6.07 | 0.52 | 1.07 |
| Hc4Ag85984 | DDC | 27.65 | 34.41 | 45.39 | 6.46 | 10.22 |
| Hc4Ag85985 | DDC | 21.1 | 28.83 | 38.81 | 6.41 | 9.71 |
| Hc4Ag85986 | DDC | 30.83 | 20.74 | 21.99 | 1.86 | 15.47 |
| Hc17Ag45963 | DDC | 0.33 | 0 | 0 | 0 | 0 |
| Hc11Cg10426 | DDC | 0.05 | 0.01 | 0.02 | 0 | 0.1 |
| Hc4Eg89409 | DDC | 57.51 | 35.8 | 49.81 | 7.67 | 17.89 |
| Hc4Eg89560 | DDC | 27.83 | 14.37 | 41.6 | 7.98 | 5.02 |
| Hc4Eg89905 | DDC | 16.41 | 10.53 | 30.88 | 2.33 | 2.31 |
| Hc4Eg89906 | DDC | 28.87 | 22.76 | 54.7 | 4.14 | 4.81 |
| Hc4Eg89907 | DDC | 16.43 | 9.95 | 16.18 | 0.97 | 4.64 |
| Hc2Bg68805 | DDC | 0.23 | 0.03 | 0.08 | 0.2 | 1.12 |
| Hc11Dg11742 | DDC | 0 | 0.09 | 0 | 0 | 0.02 |
| Hc7Dg114442 | DDC | 78.89 | 60.58 | 6.87 | 0.74 | 7.4 |
| Hc7Dg114830 | DDC | 58.95 | 32.15 | 60.52 | 16.39 | 10.22 |
| Hc11Eg13046 | DDC | 12.8 | 4.04 | 3.27 | 0.32 | 0.45 |
| Hc7Bg111274 | DDC | 79.26 | 36.47 | 7.56 | 0.71 | 10.18 |
| Hc7Bg111672 | DDC | 38.94 | 16.77 | 36.81 | 9.48 | 5.48 |
| Hc14Ag27551 | DDC | 0.09 | 0.41 | 0.71 | 2.38 | 0.45 |
| Hc16Ag39001 | DDC | 7.1 | 1.64 | 0.79 | 0.11 | 0.19 |
| Hc16Ag39672 | DDC | 11.02 | 4.61 | 37.72 | 9.33 | 2.74 |
| Hc7Cg112843 | DDC | 81.83 | 44.84 | 5.63 | 0.85 | 7.78 |
| Hc7Cg113265 | DDC | 40.22 | 38.74 | 69.19 | 14.31 | 10.47 |
| Hc7Cg113374 | DDC | 18.17 | 2.8 | 6.04 | 0.53 | 1.06 |
| Hc3Dg82828 | DDC | 7.91 | 2.1 | 3.97 | 0.81 | 0.48 |
| Hc3Dg82237 | DDC | 28.94 | 18.83 | 38.27 | 14.11 | 7.7 |
| Hc3Dg82236 | DDC | 12.46 | 15.5 | 18.45 | 4.96 | 8.18 |
| Hc3Dg82235 | DDC | 22.71 | 18.98 | 31.47 | 2.85 | 6.48 |
| Hc14Cg30690 | DDC | 0.12 | 0.04 | 0 | 0.18 | 0.07 |
| Hc14Cg30140 | DDC | 0.1 | 0.41 | 0.86 | 2.37 | 1.44 |
| Hc4Bg86492 | DDC | 44.08 | 19.27 | 16.33 | 1.73 | 7.48 |
| Hc4Bg86493 | DDC | 14.77 | 1.3 | 1.1 | 0.09 | 0.96 |
| Hc4Bg86494 | DDC | 20.35 | 8.77 | 8.37 | 0.64 | 3.19 |
| Hc4Bg86641 | DDC | 74.96 | 32.74 | 26.56 | 6.04 | 4.74 |
| Hc4Bg86990 | DDC | 39.96 | 27.77 | 56.07 | 10.79 | 6.51 |
| Hc4Bg86991 | DDC | 17.37 | 6.93 | 10.07 | 1.59 | 2.59 |
| Hc4Bg86992 | DDC | 12.73 | 10.78 | 12.46 | 0.46 | 8.29 |
| Hc8Bg118531 | DDC | 55.92 | 30.02 | 22.65 | 10.35 | 23.96 |
| Hc11Bg09133 | DDC | 4.3 | 2.78 | 1.2 | 1.64 | 1.55 |
| unanchor174g131860 | DDC | 1.07 | 1.71 | 0 | 0 | 1.05 |
| Hc16Dg43483 | NCS | 0.06 | 0 | 0 | 0 | 0 |
| Hc16Dg43484 | NCS | 505.4 | 26.96 | 20.43 | 0.08 | 0.74 |
| Hc3Ag76740 | NCS | 14.11 | 0.22 | 0.2 | 0.04 | 3 |
| Hc12Dg18970 | NCS | 2.03 | 0 | 0 | 0 | 0 |
| Hc12Dg18971 | NCS | 240.78 | 29.56 | 30.68 | 33.65 | 29.67 |
| Hc12Dg18973 | NCS | 270.54 | 33.78 | 39.32 | 2.59 | 5.27 |
| Hc3Cg82086 | NCS | 0.58 | 0.14 | 0.12 | 0.36 | 1.09 |
| Hc3Cg82084 | NCS | 9.64 | 8.75 | 8.13 | 5.97 | 15.15 |
| Hc3Cg80398 | NCS | 36.84 | 3.69 | 19.27 | 30.68 | 43.7 |
| Hc3Cg80393 | NCS | 75.54 | 0.04 | 0.09 | 0 | 1.4 |
| Hc4Dg89023 | NCS | 517.67 | 7.85 | 6.13 | 2.77 | 82.47 |
| Hc12Cg17677 | NCS | 0.06 | 0 | 0 | 0 | 0 |
| Hc7Eg116412 | NCS | 28.57 | 0 | 0 | 0 | 0 |
| Hc3Eg84976 | NCS | 6.53 | 5.66 | 5.34 | 1.06 | 5.04 |
| Hc3Eg84975 | NCS | 0.19 | 0.02 | 0 | 0 | 0 |
| Hc7Ag110071 | NCS | 1227.42 | 156.08 | 59.09 | 37.86 | 316.71 |
| Hc4Cg88061 | NCS | 11.91 | 0.23 | 0.03 | 0.09 | 0.83 |
| Hc3Bg80176 | NCS | 0.52 | 1.07 | 0.55 | 0.17 | 0.47 |
| Hc3Bg78579 | NCS | 39.26 | 0 | 0.16 | 0 | 4.69 |
| Hc4Ag86064 | NCS | 0.57 | 0 | 0 | 0 | 0.1 |
| Hc4Ag86065 | NCS | 0.95 | 0.14 | 0.05 | 0.09 | 0.44 |
| Hc11Dg12617 | NCS | 149.49 | 22.85 | 23.31 | 21.5 | 58.02 |
| Hc7Dg114815 | NCS | 246.25 | 66.1 | 23.64 | 19.95 | 87.09 |
| Hc12Ag14933 | NCS | 1.34 | 0.1 | 0.11 | 0 | 0 |
| Hc12Ag14934 | NCS | 160.92 | 11.13 | 7.63 | 0 | 0.38 |
| Hc7Bg111659 | NCS | 290.2 | 98.79 | 29.08 | 13.95 | 94.74 |
| Hc7Cg113252 | NCS | 753.13 | 147.03 | 71.74 | 53.84 | 217.8 |
| Hc3Dg82158 | NCS | 68.32 | 0.28 | 0.09 | 0 | 5.2 |
| Hc4Bg87077 | NCS | 170.2 | 1.32 | 1.07 | 0.64 | 16.73 |
| Hc11Bg09932 | NCS | 108.24 | 10.54 | 18.44 | 18.97 | 43.53 |
| unanchor680g140370 | NCS | 120.27 | 17.95 | 10.16 | 4.85 | 11.93 |
| Hc6Dg106186 | NMCH | 0.25 | 0 | 0 | 0 | 0.33 |
| Hc6Eg108095 | NMCH | 0.06 | 0 | 0.19 | 0.06 | 4.32 |
| Hc6Ag100508 | NMCH | 0.11 | 0 | 0.03 | 0 | 0.94 |
| Hc5Ag91491 | NMCH | 3.61 | 0.07 | 0.17 | 0.09 | 0.44 |
| Hc5Ag91556 | NMCH | 2.1 | 0.91 | 0.73 | 0.12 | 1.22 |
| Hc5Eg99537 | NMCH | 4.23 | 0.05 | 0.29 | 0 | 0.25 |
| Hc5Eg99600 | NMCH | 2.15 | 1.08 | 0.38 | 0.07 | 0.55 |
| Hc5Eg99601 | NMCH | 0 | 0 | 0.07 | 0.03 | 0 |
| Hc7Eg116513 | NMCH | 0.32 | 0 | 0.06 | 0 | 0.04 |
| Hc7Ag110220 | NMCH | 0.06 | 0 | 0 | 0 | 0.12 |
| Hc15Ag33344 | NMCH | 1.59 | 0 | 0.05 | 0.08 | 0.89 |
| Hc5Dg97559 | NMCH | 4.95 | 0.46 | 0.72 | 0.05 | 4.53 |
| Hc5Dg97627 | NMCH | 0 | 0 | 0 | 0.02 | 0.03 |
| Hc5Dg97628 | NMCH | 2.83 | 1.5 | 0.63 | 0.05 | 1.19 |
| Hc5Bg93472 | NMCH | 3.74 | 0.52 | 0.95 | 0.23 | 2.02 |
| Hc5Bg93533 | NMCH | 0 | 0 | 0 | 0 | 0.05 |
| Hc5Bg93534 | NMCH | 3.02 | 1.99 | 0.51 | 0.06 | 0.87 |
| Hc5Cg95531 | NMCH | 3.63 | 0 | 0.22 | 0.06 | 0.71 |
| Hc5Cg95597 | NMCH | 2.44 | 2.15 | 0.99 | 0.16 | 1.05 |
| Hc6Bg102534 | NMCH | 0 | 0.03 | 0 | 0.16 | 3.1 |
| Hc15Dg37505 | NMCH | 0.51 | 0.01 | 0.05 | 0.03 | 0.12 |
| Hc7Dg114954 | NMCH | 0 | 0 | 0 | 0 | 0.01 |
| Hc7Bg111799 | NMCH | 0 | 0 | 0 | 0 | 0.01 |
| Hc7Cg113368 | NMCH | 0 | 0 | 0 | 0 | 0.53 |
| Hc15Bg34734 | NMCH | 0.03 | 0 | 0.07 | 0 | 0.13 |
| Hc15Cg36125 | NMCH | 0.11 | 0.01 | 0.02 | 0 | 0.14 |
| unanchor1g130595 | NMCH | 15.36 | 0.9 | 0.62 | 0.92 | 2.4 |
| unanchor1g130594 | NMCH | 4.22 | 0.73 | 0.88 | 4.23 | 0.4 |
| unanchor2g133446 | NMCH | 27.81 | 4.82 | 3.49 | 11.07 | 9.93 |
| unanchor2g133445 | NMCH | 0.89 | 0.13 | 0.12 | 0.28 | 0.5 |
| unanchor3g135589 | NMCH | 7.38 | 2.82 | 0.46 | 0.87 | 3.8 |
| unanchor3g135588 | NMCH | 7.7 | 1.86 | 1.43 | 4.89 | 1.59 |
| unanchor4g137512 | NMCH | 1.94 | 1.2 | 0.12 | 0.4 | 1.37 |
| unanchor4g137511 | NMCH | 20.85 | 2.96 | 1.87 | 7.24 | 4.22 |
| unanchor5g139370 | NMCH | 6.8 | 0.98 | 0.61 | 1.04 | 1.86 |
| unanchor5g139369 | NMCH | 0.7 | 0.15 | 0.87 | 4.01 | 0.77 |
| Hc6Dg107804 | Str | 0.15 | 0.99 | 2.07 | 9 | 8.99 |
| Hc3Ag77504 | Str | 10.27 | 11.2 | 10.94 | 5.93 | 13.37 |
| Hc5Ag91032 | Str | 32.66 | 6.47 | 6.07 | 5.99 | 9.66 |
| Hc14Dg31117 | Str | 0 | 0 | 0 | 0.05 | 3.68 |
| Hc10Bg02560 | Str | 0.15 | 0 | 0.11 | 0 | 0.14 |
| Hc3Cg81196 | Str | 8.35 | 7.41 | 10.1 | 3.63 | 8.95 |
| Hc5Eg99058 | Str | 0 | 0 | 0 | 0 | 1.02 |
| Hc4Dg88220 | Str | 0.19 | 0 | 0 | 0 | 0 |
| Hc7Eg117231 | Str | 12.13 | 0.85 | 2.21 | 4.16 | 2.14 |
| Hc7Eg117562 | Str | 0 | 0 | 0 | 0 | 9.64 |
| Hc3Eg84121 | Str | 38.43 | 11.3 | 14.76 | 8.22 | 19.81 |
| Hc7Ag110880 | Str | 37.43 | 6.05 | 9.54 | 19.96 | 14.87 |
| Hc7Ag111218 | Str | 0 | 0.01 | 0.02 | 0 | 8.12 |
| Hc5Dg97083 | Str | 0 | 0.03 | 0 | 0 | 0.22 |
| Hc4Cg87271 | Str | 7.95 | 5.33 | 7.11 | 4 | 10.43 |
| Hc10Ag00915 | Str | 0 | 0.05 | 0.05 | 0 | 0 |
| Hc13Bg22781 | Str | 0 | 0 | 0 | 0 | 0.14 |
| Hc9Cg125552 | Str | 0 | 0 | 1.11 | 0.06 | 0 |
| Hc5Bg93009 | Str | 0 | 0 | 0 | 0 | 0.47 |
| Hc9Eg128497 | Str | 0 | 0 | 0.86 | 0.39 | 0.06 |
| Hc9Ag122568 | Str | 0 | 0 | 1 | 0.93 | 0.09 |
| Hc14Bg28547 | Str | 0.27 | 0 | 0.04 | 0.13 | 543.44 |
| Hc3Bg79342 | Str | 13.7 | 9.62 | 14.95 | 8.25 | 16.36 |
| Hc4Ag85279 | Str | 11.07 | 4.55 | 4.36 | 3.35 | 6.94 |
| Hc5Cg95046 | Str | 0 | 0 | 0 | 0 | 0.56 |
| Hc4Eg89200 | Str | 21.35 | 24.13 | 25.78 | 17.21 | 39.33 |
| Hc9Bg124076 | Str | 0 | 0 | 0.82 | 0 | 0 |
| Hc14Eg32425 | Str | 0.6 | 0 | 0.02 | 0.05 | 398.01 |
| Hc7Dg115624 | Str | 25.27 | 11.55 | 18.01 | 22.18 | 14.87 |
| Hc7Dg115948 | Str | 0 | 0.1 | 0.05 | 0.16 | 7.23 |
| Hc7Bg112446 | Str | 23.99 | 4.07 | 7.2 | 11.41 | 6.6 |
| Hc7Bg112448 | Str | 0 | 0 | 0 | 0.09 | 0.08 |
| Hc7Bg112768 | Str | 0.15 | 0.11 | 0.04 | 0.21 | 6.34 |
| Hc14Ag27263 | Str | 0.05 | 0 | 0 | 0.02 | 143.65 |
| Hc7Cg114057 | Str | 29.17 | 5.76 | 12.18 | 20.9 | 14.13 |
| Hc7Cg114366 | Str | 0 | 0 | 0 | 0 | 17.12 |
| Hc10Dg05551 | Str | 0 | 0 | 0.08 | 0 | 0.04 |
| Hc3Dg82920 | Str | 11.12 | 5.75 | 8.59 | 4.43 | 9.08 |
| Hc14Cg29841 | Str | 0.43 | 0 | 0 | 0.04 | 524.37 |
| Hc10Cg04077 | Str | 0.06 | 0.03 | 0.17 | 0 | 0 |
| Hc10Eg07044 | Str | 0 | 0 | 0.08 | 0 | 0.12 |
| unanchor2g132425 | Str | 0 | 0 | 0 | 0 | 0.34 |
| unanchor5g138350 | Str | 0 | 0 | 0 | 0 | 0.21 |
| Hc16Bg41167 | TYR | 0.19 | 0 | 0.19 | 0.03 | 0 |
| Hc11Ag08617 | TYR | 287.92 | 322.2 | 337.72 | 117.86 | 522.22 |
| Hc11Ag08618 | TYR | 30.22 | 18.03 | 21.01 | 8.39 | 9.4 |
| Hc11Ag08622 | TYR | 7.47 | 36.05 | 185.27 | 88.15 | 165.92 |
| Hc15Ag34254 | TYR | 6.82 | 6.23 | 11.92 | 23.09 | 7.11 |
| Hc9Cg125045 | TYR | 19.2 | 26.37 | 20.93 | 18.21 | 26.02 |
| Hc9Ag122060 | TYR | 11.57 | 23.83 | 16.7 | 12 | 22.77 |
| Hc11Cg11225 | TYR | 100.72 | 222.32 | 217.89 | 72.76 | 317.84 |
| Hc11Cg11227 | TYR | 12.91 | 9.16 | 13.01 | 4.42 | 6.69 |
| Hc11Cg11230 | TYR | 4.89 | 21.2 | 148.18 | 84.4 | 123.2 |
| Hc13Eg25759 | TYR | 6.63 | 0.19 | 3.85 | 10.26 | 0.82 |
| Hc12Eg20560 | TYR | 10.74 | 0.12 | 1.94 | 6.35 | 0.76 |
| Hc9Bg123566 | TYR | 10.67 | 16.11 | 10.81 | 6.54 | 14.35 |
| Hc15Dg38417 | TYR | 12.76 | 20.79 | 67.05 | 77.98 | 29.88 |
| Hc11Dg12615 | TYR | 4.44 | 47.48 | 111.36 | 56.88 | 109.18 |
| Hc11Dg12618 | TYR | 3.22 | 0.43 | 2.63 | 1.29 | 1.73 |
| Hc11Dg12620 | TYR | 62.97 | 166.46 | 145.87 | 49.8 | 184.53 |
| Hc11Eg13857 | TYR | 0 | 29.5 | 39.53 | 7.82 | 54.8 |
| Hc11Eg13859 | TYR | 0 | 0.57 | 1.7 | 1.26 | 2.45 |
| Hc11Eg13862 | TYR | 3.77 | 43.32 | 104.61 | 60.5 | 89.05 |
| Hc12Ag14931 | TYR | 0 | 0 | 0.03 | 1.02 | 0 |
| Hc15Bg35638 | TYR | 27.57 | 31.54 | 35.75 | 53.36 | 26.45 |
| Hc15Cg37026 | TYR | 11.41 | 14.74 | 47.05 | 58.78 | 13.2 |
| Hc11Bg09929 | TYR | 83.29 | 159.71 | 207.91 | 56.59 | 261.24 |
| Hc11Bg09931 | TYR | 29.91 | 6.23 | 14.83 | 11.84 | 11.45 |
| Hc11Bg09934 | TYR | 7.5 | 61.59 | 177.66 | 185.03 | 307.67 |
| unanchor443g138171 | TYR | 4.04 | 0.9 | 11.53 | 18.71 | 1.7 |
| unanchor680g140367 | TYR | 0 | 29.5 | 39.53 | 7.82 | 54.8 |
| unanchor680g140369 | TYR | 28.21 | 5.52 | 4.79 | 1.37 | 2.67 |
| unanchor921g140930 | TYR | 69.39 | 65.01 | 77.33 | 19.09 | 137.1 |
| unanchor1048g131201 | TYR | 0 | 29.5 | 39.53 | 7.82 | 54.8 |
